# Enteric tuft cells coordinate timely expulsion of the tapeworm *Hymenolepis diminuta* from the murine host by coordinating local but not systemic immunity

**DOI:** 10.1371/journal.ppat.1012381

**Published:** 2024-07-31

**Authors:** Sruthi Rajeev, ShuHua Li, Aralia Leon-Coria, Arthur Wang, Lucas Kraemer, Susan Joanne Wang, Annaliese Boim, Kyle Flannigan, Adam Shute, Cristiane H. Baggio, Blanca E. Callejas, Wallace K. MacNaughton, Constance A. M. Finney, Derek M. McKay

**Affiliations:** 1 Gastrointestinal Research Group and Inflammation Research Network, Department of Physiology and Pharmacology, Calvin, Phoebe and Joan Snyder Institute for Chronic Diseases, Cumming School of Medicine, University of Calgary, Calgary, Alberta, Canada; 2 Host-Parasite Interactions Program, University of Calgary, Calgary, Alberta, Canada; 3 Department of Biology, Faculty of Science, University of Calgary, Calgary, Alberta, Canada; New York University, UNITED STATES OF AMERICA

## Abstract

Recognizing that enteric tuft cells can signal the presence of nematode parasites, we investigated whether tuft cells are required for the expulsion of the cestode, *Hymenolepis diminuta*, from the non-permissive mouse host, and in concomitant anti-helminthic responses. BALB/c and C57BL/6 mice infected with *H*. *diminuta* expelled the worms by 11 days post-infection (dpi) and displayed DCLK1^+^ (doublecortin-like kinase 1) tuft cell hyperplasia in the small intestine (not the colon) at 11 dpi. This tuft cell hyperplasia was dependent on IL-4R*α* signalling and adaptive immunity, but not the microbiota. Expulsion of *H*. *diminuta* was slowed until at least 14 dpi, but not negated, in tuft cell-deficient *Pou2f3*^*-/-*^ mice and was accompanied by delayed goblet cell hyperplasia and slowed small bowel transit. Worm antigen and mitogen evoked production of IL-4 and IL-10 by splenocytes from wild-type and *Pou2f3*^*-/-*^ mice was not appreciably different, suggesting similar systemic immune reactivity to infection with *H*. *diminuta*. Wild-type and *Pou2f3*^*-/-*^ mice infected with *H*. *diminuta* displayed partial protection against subsequent infection with the nematode *Heligmosomoides bakeri*. We speculate that, with respect to *H*. *diminuta*, enteric tuft cells are important for local immune events driving the rapidity of *H*. *diminuta* expulsion but are not critical in initiating or sustaining systemic Th2 responses that provide concomitant immunity against secondary infection with *H*. *bakeri*.

## Author summary

The small intestinal tuft cell is emerging as a versatile sentinel cell type in a variety of infections involving protozoans, nematodes, and certain bacteria. Whether the tuft cell responds to enteric cestode infections and the extent of its involvement in mediating the anti-helminth response is not yet characterized. Using mice infected with *Hymenolepis diminuta*, we show that tuft cells help accelerate expulsion of this lumen-dwelling cestode, by coordinating local events that form an integral part of the classical “weep and sweep” anti-helminthic response. We further show that although infection with *H*. *diminuta* induces both tuft cell hyperplasia as well as protection from subsequent infection with the nematode *Heligmosmoides bakeri*, the tuft cell is not solely responsible for mediating systemic and other local Th2 responses against *H*. *diminuta*. Our work reveals subtle roles for the tuft cell in initiating and coordinating host anti-parasitic activity in cestode infections. At the same time, we show that redundancies exist in the host’s mucosal arsenal that ultimately mediate worm expulsion in tuft cell deficient mice.

## Introduction

Helminths have evolved to avoid or dampen host defences to ensure their survival and reproduction [1]. Conversely, hosts have co-evolved to mount a multi-pronged approach to eliminate parasites, deploying innate and adaptive immunity to varying degrees, depending on how the parasite is detected [2]. Consequently, analyses of laboratory-based helminth-rodent models provide valuable insights on diversity in helminth life cycles, niche selection, and nuances in the host’s anti-helminth immunity. With respect to enteric parasites, longstanding knowledge gaps include how the helminth is detected and the role(s) for epithelial cell subtypes in worm clearance from the gut [3–5].

Tuft cells are of the taste receptor cell lineage and express a repertoire of chemosensory receptors and ion channels such as the bitter taste receptor, TAS2R, the succinate receptor, SUCNR1, and the cation channel, TRPM5 (transient receptor potential cation channel subfamily M member 5) [3,6,7]. Small intestinal tuft cell hyperplasia has been observed following infection with nematodes (*Nippostrongylus brasiliensis*, *Trichinella spiralis*, *Heligmosmoides polygyrus (bakeri)*, *Teladorsagia circumcincta*, *Haemonchus contortus*), the trematode, *Echinococcus caproni*, the protozoans, *Tritrichomonads* and the cestode, *Hymenolepis microstoma* [3–5,8–10]. Tuft cell production of interleukin (IL)-25, prostaglandins, and cysteinyl leukotrienes [5,11–13] can activate innate lymphoid cells 2 (ILC2) (and likely other mucosal cells) to amplify and maintain Th2 immunity against *N*. *brasiliensis* and *H*. *polygyrus* in the murine host [3,5]. Analyses are revealing pathogen specificity in the role of tuft cells, where, for example, leukotriene release was found to be important for expulsion of *N*. *brasiliensis* but not *Tritrichomonads* [11]. Furthermore, the ligands that tuft cells sense may differ between parasitic infections [14], warranting a thorough understanding of whether tuft cell responses are critical in the host response to all classes of intestinal helminths.

*H*. *diminuta* is a cestode that has no abrasive hooks, teeth or an alimentary canal. During its life cycle, *H*. *diminuta* neither migrates outside the gastrointestinal tract of the host (infection is by ingestion), nor invades the enteric epithelium. Mice, unlike rats (permissive hosts of *H*. *diminuta*), expel this lumen-dwelling parasite by 8–11 days post-infection (dpi) [15]. Compared to other helminths such as the nematode *N*. *brasiliensis*, whose larvae migrate to the lung before entering the gut or *H*. *polygyrus*, which invades the enteric epithelium during larval stages, *H*. *diminuta* may present a different gamut of stimuli in its mouse host [16].

To advance knowledge of the putative functional importance of tuft cells against cestode infections, experiments were performed to determine if these cells are critical in the expulsion of *H*. *diminuta* from mice, and whether any *H*. *diminuta*-evoked tuft cell response affected the outcome of subsequent infection with the unrelated nematode, *H*. *bakeri* [17,18]. Our data reveal that tuft cells coordinate the rapid expulsion of *H*. *diminuta* and that tuft cell deficiency did not abrogate concomitant immunity against secondary infection with *H*. *bakeri*. Despite the delayed kinetics of expulsion of *H*. *diminuta* and local immune-deficiencies, infected *Pou2f3*^*-/-*^mice, which are tuft cell deficient, displayed increased splenocyte production of IL-4, IL-13, and IL-10 (i.e., systemic Th2-immunity) similar to wild type (WT) littermates. Thus, tuft cells are important but not essential in the clearance of *H*. *diminuta* from its mouse host and primarily mediate local but not systemic defences against the parasite.

## Results

### Infection with *H*. *diminuta* induces small intestinal but not colonic tuft cell hyperplasia in BALB/c mice

**Fig 1:**
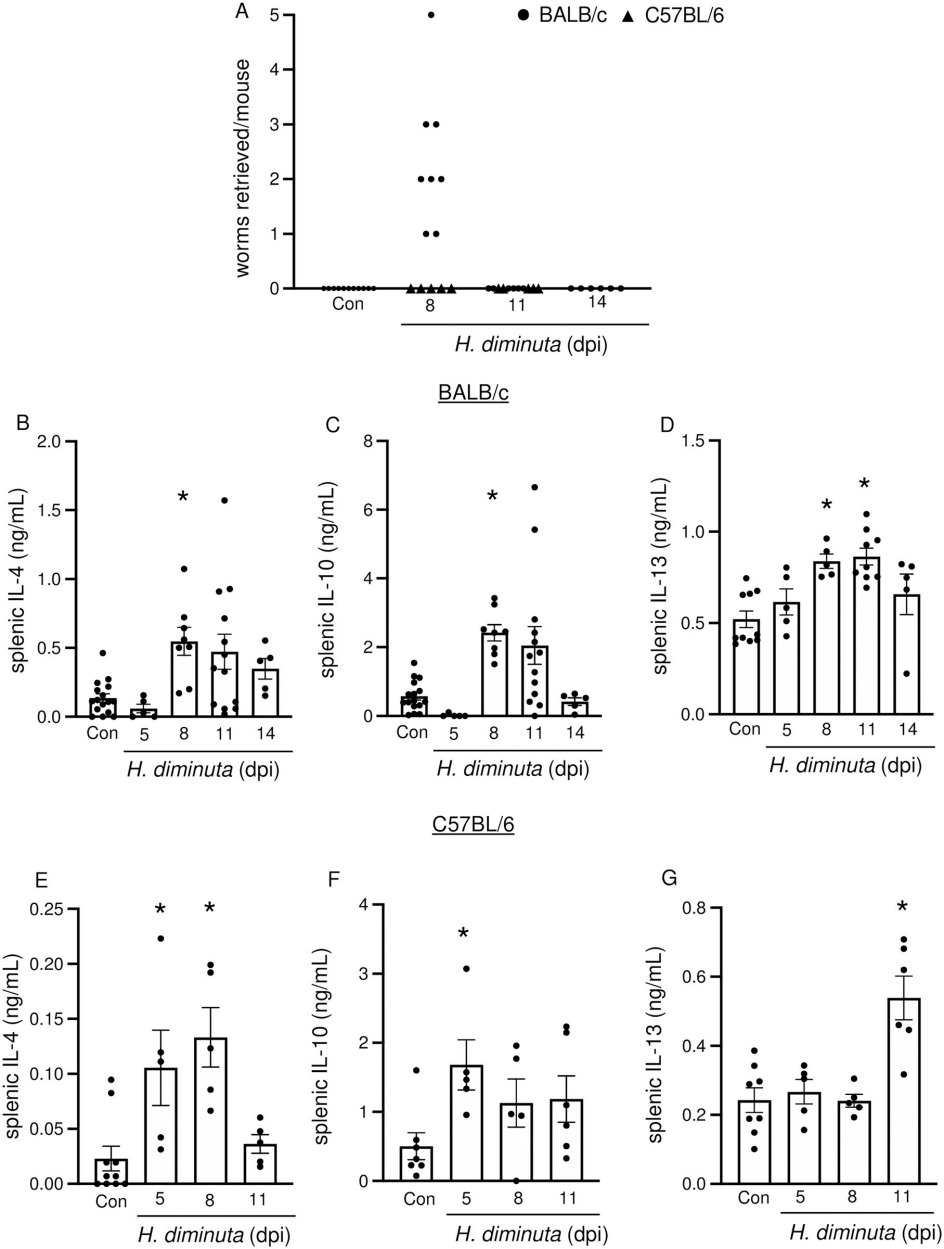
*H*. *diminuta*-infection is cleared by 11 days post-infection and induces systemic Th2 cytokines in the murine host. Male BALB/c and C57BL/6 mice were infected with 5 cysticercoids of *H*. *diminuta* and assessed at days post-infection (dpi). **(A)** Murine small intestines were flushed with ice cold PBS to retrieve and enumerate worms. Cytokine ELISAs were performed on supernatants from splenic cells (5x10^6^/mL) from **(B, C, D)** BALB/c and **(E, F, G)** C57BL/6 mice stimulated with concanavalin A (2 μg/mL, 48h). Data are mean ± SEM, n = 5-16/group, pooled from 2–3 experiments, * *p*<0.05 compared to the control (Con) group, analysed by Kruskal Wallis test with Dunn’s test for multiple comparisons.

The use of transgenic mice of different genetic backgrounds in this study prompted comparison of *H*. *diminuta*-infection in both BALB/c and C57BL/6 mice. Despite BALB/c mice being Th2-predisposed, male C57BL/6 mice expelled *H*. *diminuta* quicker than BALB/c: *H*. *diminuta* was not apparent in luminal flushes from BALB/c mice by 11 dpi, whereas expulsion was complete by 8 dpi in C57BL/6 mice (n = 5) (Fig 1A) [19]. *H*. *diminuta* worms can be challenging to detect in luminal flushes under a dissection microscope at 5 dpi due to their small size and cream colour, but were visualized in small intestinal H&E sections at this time point (S1A Fig), confirming that the worm excysts in C57BL/6 mice [20] (also see cytokine data). As a surrogate of host response to infection with *H*. *diminuta*, infected mice showed increased concanavalin-A (con-A) stimulated splenic production of IL-4, IL-10, and IL-13 (Fig 1B–1G), the variability in the data reflecting the individual mouse’s response to the worm.

**Fig 2:**
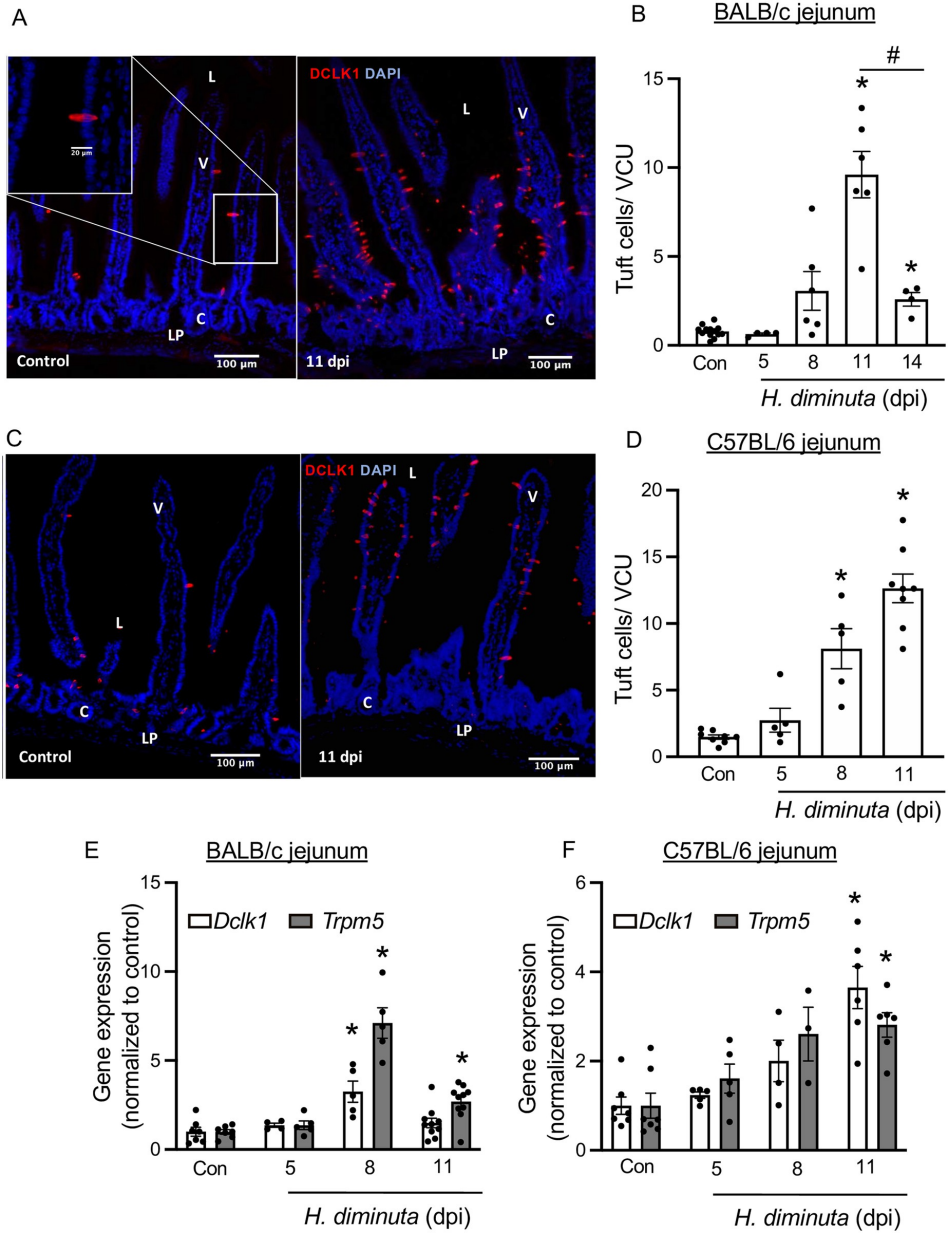
*H*. *diminuta*-infection induces murine small intestinal tuft cell hyperplasia. Male BALB/c and C57BL/6 mice were infected with 5 cysticercoids of *H*. *diminuta* and assessed at days post-infection (dpi). **(A, C)** Representative images of mid-jejunal cryosections (10 μm) from control and infected BALB/c and C57BL/6 mice (11 dpi) immunostained for DCLK1 (red) and counterstained with DAPI (blue). “L”–lumen, “V”- villus,” C”- crypt, “LP”- lamina propria and “S”- serosa on the image. DCLK1^+^ cells were enumerated per villus crypt unit (VCU) and averaged over 20 respective units per mouse. **(E, F)** Relative mRNA expression (compared to the 18S rRNA housekeeping gene) in mid-jejunal segments from BALB/c and C57BL/6 mice respectively, was assessed by real-time PCR. Data are mean ± SEM values, n = 4-9/group, pooled from 2–3 experiments, * *p*<0.05 compared to the control (Con) group, analysed by **(B, D)** Browns Forsythe and Welch’s ANOVA and Dunnett’s test or **(E, F)** Kruskal Wallis test and Dunn’s test for multiple comparisons; # *p*<0.05 comparing tuft cell counts at 14 dpi vs at 11 dpi analysed by Welch’s *t* test **(A)**.

Infection with *H*. *diminuta* resulted in significant (p<0.05) ~12-fold and ~8-fold increases in jejunal doublecortin-like kinase-1^+^ (DCLK1^+^) epithelial tuft cells in BALB/c and C57BL/6 mice, respectively at 11 dpi (Fig 2A–2D). BALB/c ileum also revealed increased numbers of tuft cells (S1B Fig). In contrast, infection with *H*. *diminuta* did not result in a change in the number of DCLK1^+^ tuft cells in the colon of BALB/c or C57BL/6 mice (S1C–S1E Fig), prompting a focus on small intestinal tuft cells in subsequent studies. Supporting the immunolocalization studies, qPCR revealed increased expression of the tuft cell associated genes, *Dclk1* and *Trpm5* mRNA between 8–11 dpi in jejunal tissue excised from *H*. *diminuta*-infected BALB/c and C57BL/6 mice, respectively (Fig 2E and 2F). Examination of the permissive rat host revealed mature, gravid *H*. *diminuta* in all the rats (S2A–S2C Fig). Immunostaining of jejunal sections from rats infected 3–6 months previously revealed a ~2.5-fold statistically significant increase in DCLK1^+^ tuft cells compared to uninfected controls (S2D Fig).

### *H*. *diminuta* evoked tuft cell hyperplasia is IL-4Rα and adaptive immunity dependent

**Fig 3:**
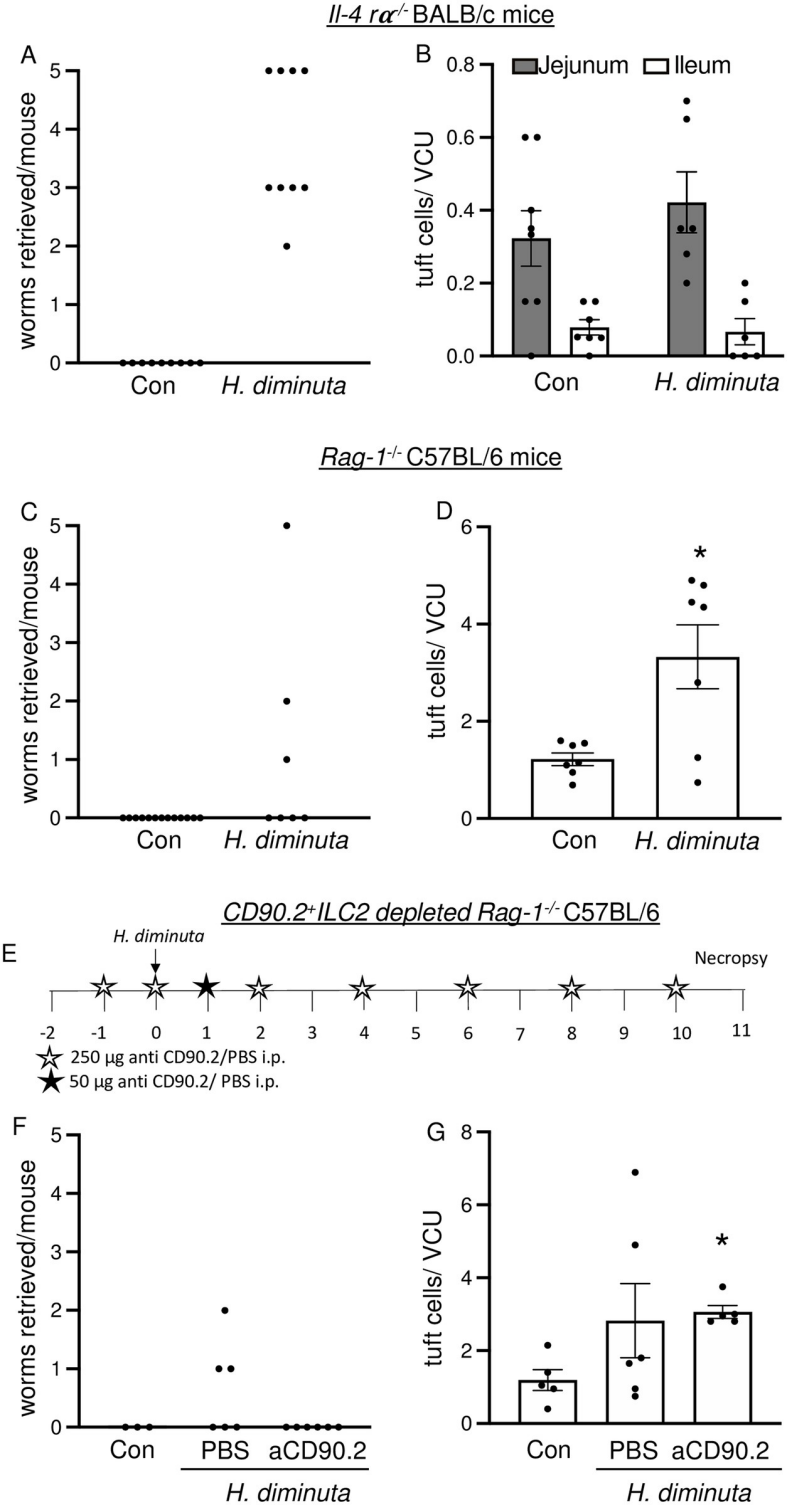
Tuft cell hyperplasia in response to *H*. *diminuta* infection depends on IL-4Rα signalling and adaptive immunity. Male *Il-4rα*^-/-^ **(A, B)** and *Rag-1*^-/-^ mice (with and without anti-CD90.2 antibody treatment) **(C-G)** were infected with 5 cysticercoids of *H*. *diminuta* and assessed at 11 days post-infection (dpi). For ILC2 depletion, *Rag-1*^-/-^ mice were treated with 1800 μg anti-CD90.2 antibody as shown in **(E)**. **(A, C, F)** Small intestines were flushed with ice cold PBS at necropsy to retrieve and enumerate worms **(B, D, G)** Mid-jejunal and **(B)** ileal cryosections were immunostained with anti-DCLK1 antibody and counterstained with DAPI. DCLK1^+^ cells were enumerated per villus crypt unit (VCU). Data are mean ± SEM values, n = 3-9/group, pooled from 2–3 experiments, * *p*<0.05 compared to control (Con) mice and analysed by **(B)** Welch’s unpaired *t* test or **(D, G)** Browns Forsythe and Welch’s ANOVA test and Dunnet’s post-test for multiple comparisons.

*Il-4rα*^-/-^ BALB/c mice do not expel *H*. *diminuta* (Fig 3A) [19]. These mice have significantly reduced jejunal and ileal tuft cell numbers at homeostasis compared to WT mice, and infection with *H*. *diminuta* did not elicit tuft cell hyperplasia (Fig 3B compared to Figs 2B and S1B).

Expulsion of *H*. *diminuta* from *Rag1*^*-/-*^ mice was slowed but not negated, with 3 of 7 mice harbouring worms at 11 dpi (Fig 3C). The jejunal tuft cell hyperplasia apparent in infected wild-type C57BL/6 mice (Fig 2D) was significantly blunted in *H*. *diminuta*-infected male C57BL/6 *Rag1*^*-/-*^ mice (Fig 3D), indicative of a major role of T and/or B cells via IL-4/IL-13 signalling in the hyperplasia response. To investigate if ILC2s were responsible for the residual tuft cell hyperplasia seen in *H*. *diminuta*-infected *Rag1*^*-/-*^ mice, Lin^-^CD90.2^+^GATA3^+^ ILC2 cells were depleted by anti-CD90.2 antibody treatment (Figs 3E and S3). *Rag1*^*-/-*^CD90.2^+^ILC2-depleted mice were able to expel *H*. *diminuta* and the degree of jejunal tuft cell hyperplasia was not appreciably different from *Rag-1*^*-/-*^ mice at 11 dpi (Fig 3F and 3G). These data suggest that CD90.2^+^ ILC2s do not contribute to the ~3-fold increase in jejunal tuft cells observed in *H*. *diminuta*-infected *Rag1*^*-/-*^ mice at 11 dpi. However, the CD90.2 antibody treatment did not deplete CD90.2^- /low^Lin^-^Gata3^+^ cells (S3A–S3C Fig), indicating that either this subset of ILC2s or other innate immune cells may contribute to tuft cell hyperplasia in the *Rag1*^*-/-*^ mouse.

Infected *Rag1*^*-/-*^ mice treated with anti-CD90.2 antibodies display jejunal eosinophilia (SiglecF^+^ immune cells) in the lamina propria (S4 Fig). To test if depletion of eosinophils abrogates tuft cell hyperplasia, *Rag-1*^-/-^ mice were treated with a regime of anti-CD90.2 and anti-IL-5 antibodies (S5A Fig). Anti-IL-5 antibody treatment depleted blood eosinophils (S5B Fig) and did not alter worm expulsion dynamics when compared to isotype controls (S5C Fig). However, there was no statistically significant difference in jejunal tuft cell numbers when tissue from infected mice treated with anti-CD90.2 antibody and anti-CD90.2/anti-IL-5 antibody was compared (S5D Fig and compared to Fig 3G).

### *H*. *diminuta*-infection induced tuft cell hyperplasia is independent of the microbiome

**Fig 4:**
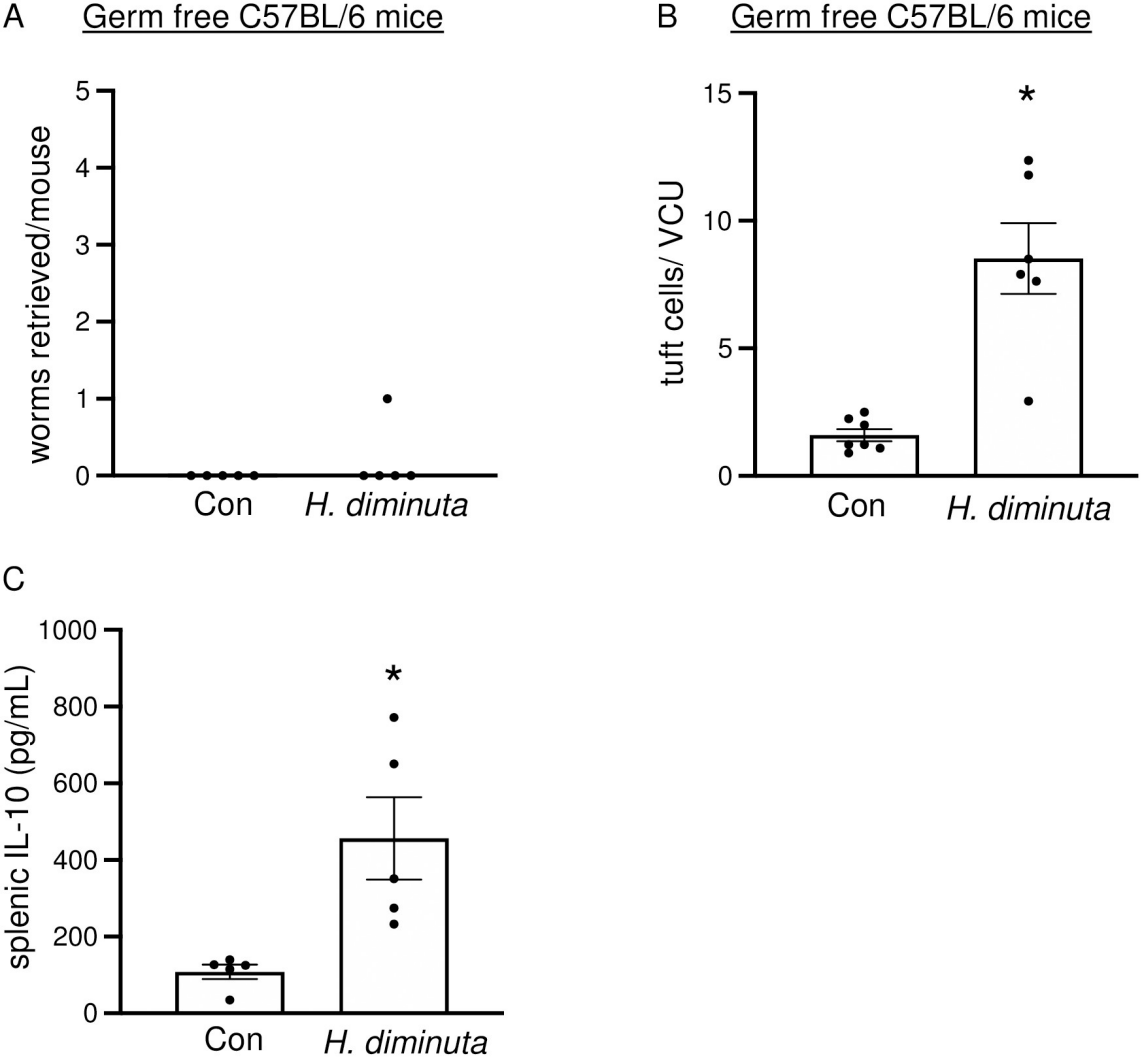
Tuft cell hyperplasia in response to *H*. *diminuta* does not depend on the microbiome. Male germ free C57BL/6 mice were infected with 10 antibiotic-treated cysticercoids of *H*. *diminuta* and assessed at 11 days post-infection (dpi). **(A)** Murine small intestines were flushed with ice cold PBS at necropsy to retrieve and enumerate worms. **(B)** Mid-jejunal sections were immunostained with anti-DCLK1 antibody and counterstained with DAPI. DCLK1^+^ cells were enumerated per villus crypt-unit (VCU) averaged over at least 3 random VCUs/mouse. **(C)** IL-10 ELISA was performed on supernatants from splenic cells (5x10^6^/mL) stimulated with concanavalin A (2 μg/mL, 48h). Data are mean ± SEM values, n = 5-8/group, pooled from 2 experiments, * *p*<0.05 compared to control (Con) mice and analysed by Welch’s unpaired *t* test.

Infection with *H*. *diminuta* modulates the rodent hosts’ colonic microbiota [19]. Germ free C57BL/6 mice expelled *H*. *diminuta* by 11 dpi (Fig 4A) and these mice displayed jejunal tuft cell hyperplasia at 11 dpi (Fig 4B). The mice showed elevated splenic IL-10 at 11 dpi (Fig 4C), supporting published literature [19] that germ free mice display elevated type 2 cytokines in response to infection with *H*. *diminuta*. In these mice, at this time point splenocyte production of IL-4 and IL-13 in response to ConA was below the level of assay detection.

### Tuft cell deficiency delays but does not abrogate expulsion of *H*. *diminuta* from mice

**Fig 5:**
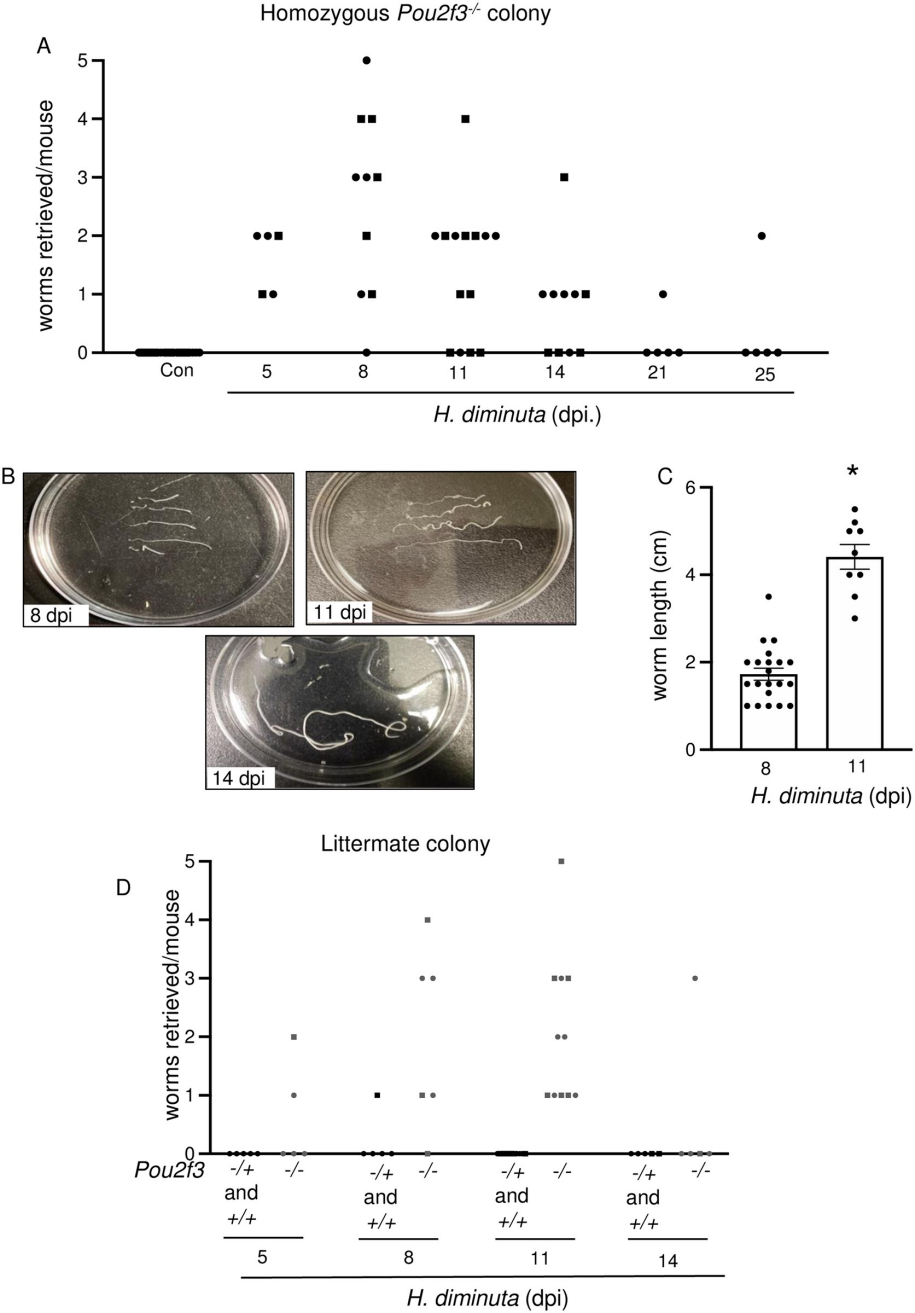
Tuft cell deficiency delays expulsion of *H*. *diminuta* from the murine intestine. Male (●) and female (◼) *Pou2f3*^*-/-*^ (homozygous colony) **(A-C)**, and *Pou2f3*^+/+, +/-, -/-^ littermate mice **(D)** were infected with 5 cysticercoids of *H*. *diminuta* and assessed at days post-infection (dpi). **(A, B)** Small intestines were flushed with ice cold PBS to retrieve and enumerate worms. **(C)** *H*. *diminuta* worms from *Pou2f3*^-/-^ mice were significantly longer at 11 dpi. **(D)** Unlike their infected wild type littermates that show no detectable worms, *Pou2f3*^-/-^ mice still harbor *H*. *diminuta* worms at 11 dpi. Data in **(C)** are mean ± SEM, n = 9-21/group, pooled from 2–3 experiments, *p<0.05 analysed by Welch’s *t* test.

*Pou2f3*^*-/-*^ mice lack tuft cells [3] and are, therefore, a useful model to assess tuft cell function. Mice from a homozygous *Pou2f3*^*-/-*^ colony infected with *H*. *diminuta* displayed a significant delay in worm expulsion, with 66% and 50% of the mice harboring worms at 11 and 14 dpi, respectively (Fig 5A–5C); time-points when *H*. *diminuta* is not found in the gut of WT mice (Fig 1A). As late as 21–25 dpi, 1 of 5 *Pou2f3*^*-/-*^ mice harboured *H*. *diminuta* (Fig 5A). A previous study using broad-spectrum antibiotic-treatment and germ-free mice indicated that the gut bacteria does not influence the expulsion of *H*. *diminuta* from WT BALB/c or C57BL/6 mice [19]. 16S analysis of the fecal microbiome of commercially sourced C57BL/6 mice and the in-house *Pou2f3*^-/-^ mice revealed a difference in α diversity (Observed and Chao1 measures) and β diversity between the two groups (S6 and S7 Figs). To control for any contribution from the gut microbiota, the previous data from a homozygous *Pou2f3*^-/-^ colony were compared with *Pou2f3*^*-/-*^ mice from *Pou2f3*^*-/+*^*-*X-*Pou2f3*^*-/+*^ breeding pairs. Comparing littermates confirmed the delay in expulsion of *H*. *diminuta* from the tuft cell deficient mice, where worms were retrieved from all infected *Pou2f3*^*-/-*^ mice at 11 dpi (Fig 5D). Additionally, 16S sequencing indicate that mice bred at Univ. Calgary have similar α-diversity regardless of genotype or parents (S6 and S7 Figs).

### Tuft cell-deficient mice display deficiencies in local immune effector responses compared to WT littermates

**Fig 6:**
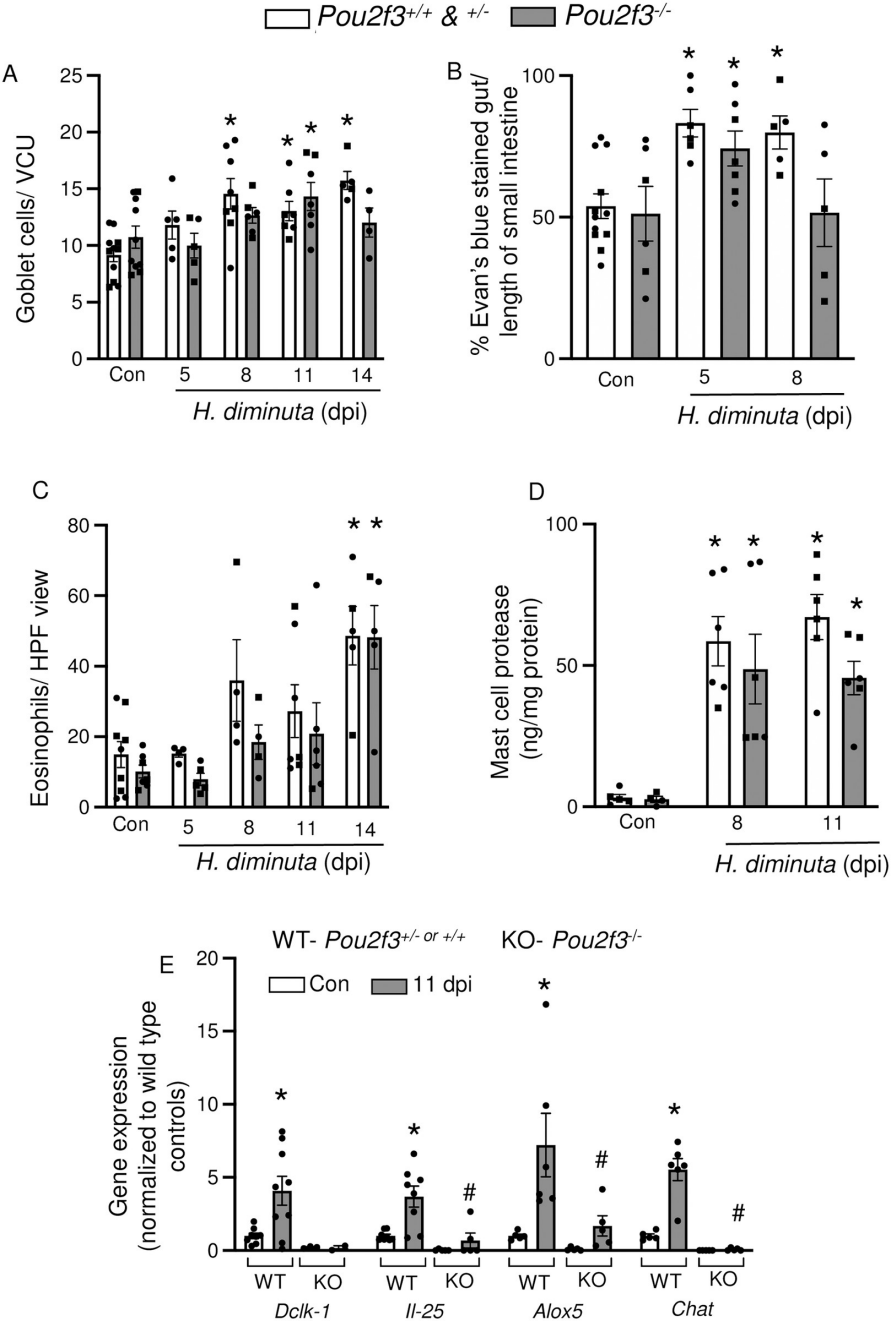
Tuft cell-deficient mice display local deficiencies in response against *H*. *diminuta*. Male (●) and female (◼) littermate *Pou2f3*^+/+, +/-, -/-^ mice were infected with 5 cysticercoids of *H*. *diminuta* and assessed at days post-infection (dpi). **(A)** Goblet cells (PAS^+^) were enumerated in mid-jejunal sections and are represented as number of cells/villus crypt unit (VCU) averaged over at least 3 full VCUs/mouse. **(B)** Small intestinal transit was measured as % of the small intestine covered by Evan’s blue dye 15 min after oral gavage. **(C)** Eosinophils were enumerated in mid-jejunal sections and presented as cells per HPF. **(D)** Mast cell protease-1 concentrations were measured by ELISA in mid-jejunal homogenates. **(E)** Small intestinal epithelial cell isolations from naïve mice and *H*. *diminuta*-infected mice (11 dpi) were used for mRNA analysis. Gene expression data were normalized within each sample to the housekeeping gene 18S rRNA and then to uninfected wild type mice. Data are mean ± SEM, n = 2-9/group, pooled from 2–3 experiments, **(A-D)** * represents *p*<0.05 compared to uninfected mice and **(E)** * and # represent *p*<0.05 analysed by two-way ANOVA and Tukey’s post-test, where * compared to uninfected mice of the respective genotype and # compared to infected wild type (WT) control.

Expulsion of enteric helminths consists of the coordinated action of multiple local effector mechanisms including mucus production, increased movement of water into the lumen, and smooth muscle contractility [15,21]. Infection with *H*. *diminuta* resulted in a significant (p<0.05) increase in jejunal PAS^+^ goblet cells at 8, 11 and 14 dpi (Fig 6A) in WT mice, but a delayed increase in goblet cells at 11 dpi in *Pou2f3*^*-/-*^ littermates. Small intestinal motility defined by Evan’s blue transit was increased in WT and *Pou2f3*^-/-^ mice at 5 dpi but had returned to baseline levels by 8 dpi in the tuft cell deficient animals (Fig 6B). Large intestinal motility was not affected by *H*. *diminuta* in either mouse genotype and baseline jejunal short-circuit current (Isc) and stimulated Isc responses were not statistically different between naïve and infected *Pou2f3*^*+/-*^ and *Pou2f3*^*-/-*^ littermates (S1 Table).

Multiple helminths, including *H*. *diminuta*, evoke gut and blood eosinophilia and mastocytosis around the time of worm expulsion from immunocompetent mice [22]. Jejunum from both infected *Pou2f3*^*+/-*^ and *Pou2f3*^*-/-*^ mice had increased eosinophils at 14 dpi (Fig 6C). Mast cell activity assessed by jejunal levels of mast cell protease-1 (MCPT-1) was similarly elevated in *Pou2f3*^*+/-*^ and *Pou2f3*^*-/-*^ mice at 8 and 11 dpi (Fig 6D).

qPCR analysis of epithelial isolates revealed that infected *Pou2f3*^*-/-*^ mice showed drastically reduced mRNA expression for genes associated with common tuft cell mediators: 5-lipooxygenase (*Alox5*), choline acetyltransferase (*Chat*) and interleukin-25 (*Il-25*) (Fig 6E) as well as *Dclk1*, the tuft cell marker, in comparison to infected *Pou2f3*^*+/-*^ mice.

### Tuft cell deficient mice display similar systemic immune responses compared to wild type littermates

**Fig 7:**
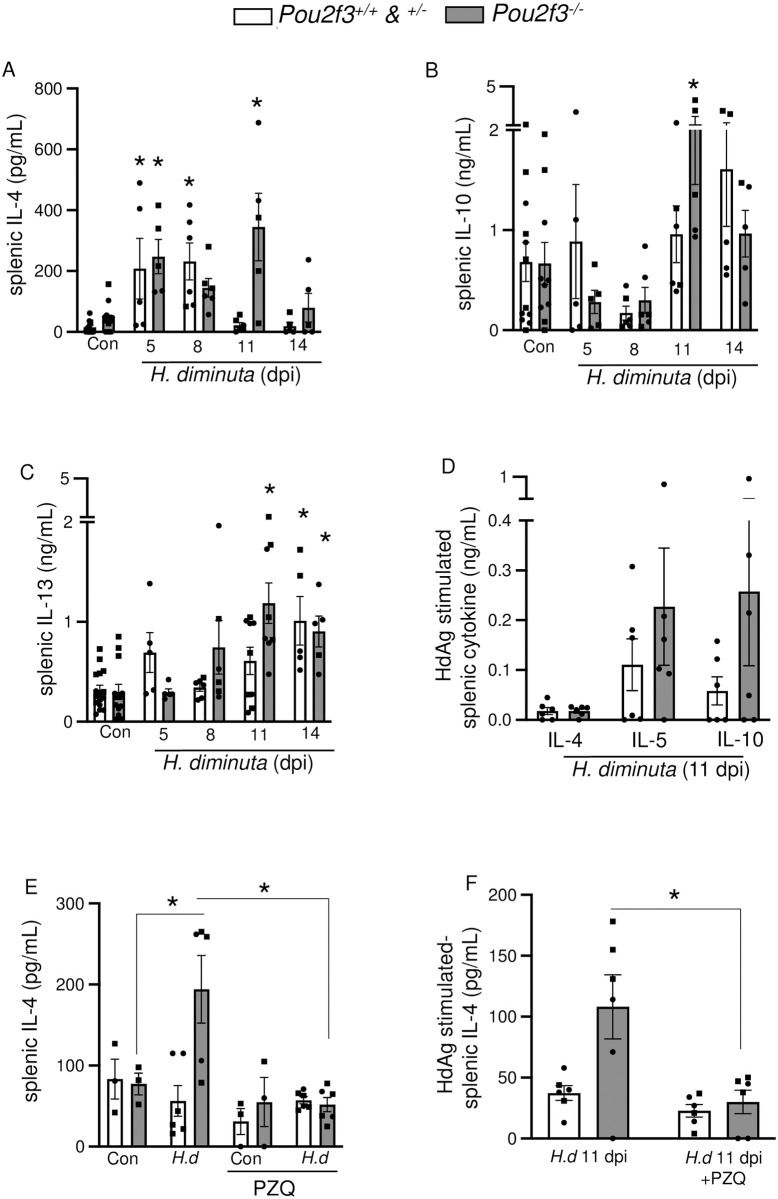
Tuft cell-deficient mice display systemic Th2 immune response following infection with *H*. *diminuta*. Male (●) and female (◼) *Pou2f3*^+/+, +/-, -/-^ mice were infected with 5 cysticercoids of *H*. *diminuta (H*.*d)* and assessed at days post-infection (dpi). In **(E, F)**, mice were treated with praziquantel (PZQ; 1 mg/mouse by oral gavage) at 8 dpi prior to necropsy on 11 dpi. **(A, B, C, E)** Cytokine ELISAs for IL-4, IL-10, and IL-13 were performed on supernatants from splenic cells (5x10^6^) stimulated with concanavalin A (2 μg/mL, 48h) or **(D, F)** a PBS-soluble crude extract of adult *H*. *diminuta* (HdAg, 200 μg/mL, 96h). Data are mean ± SEM, n = 3-6/group, pooled from 1–3 experiments, * *p*<0.05 compared to control (Con) uninfected mice, or as indicated on the graph, analysed by **(A, B, C)** Two-Way ANOVA and Dunnet’s/ Tukey’s post-test or **(D)** multiple Welch’s *t* tests.

Con-A stimulated splenocytes from *Pou2f3*^*-/-*^ mice show increased production of IL-4 at 5 dpi, similar to that from their *Pou2f3*^*+/+*^/*Pou2f3*^*+/-*^ littermates, and still had elevated IL-4 and IL-10 levels at 11 dpi (Figs 7A, 7B and S7). ConA stimulated splenic IL-13 was elevated in *Pou2f3*^*-/-*^ mice at 11 dpi and both genotypes by 14 dpi (Fig 7C). Challenge of splenocytes from *Pou2f3*^-/-^ and *Pou2f3*^+/-^ mice at 11 dpi with *H*. *diminuta* antigen for 96h resulted in IL-4, IL-5 and IL-10 synthesis (Fig 7D), which was negligible in cells from uninfected mice of either genotype (S1 Data), confirming systemic immunity to *H*. *diminuta* in the *Pou2f3*^*-/-*^ mice. To test if the elevated systemic responses in *Pou2f3*^*-/-*^ mice were driven by the presence of a worm burden at 11 dpi (Fig 5D), mice were treated with the anti-helminthic praziquantel at 8 dpi and systemic cytokine responses were tested at 11 dpi (Figs 7E, 7F and S9A Fig). *H*. *diminuta* infected, praziquantel treated *Pou2f3*^*-/-*^ mice had significantly reduced worm burden (S9B Fig) and produced lower levels of splenic IL-4 compared to infected controls that did not receive praziquantel (Fig 7E and 7F). *Pou2f3*^*+/+*^, but not *Pou2f3*^-/-^, mice infected with *H*. *diminuta* showed significant blood eosinophilia, which was not affected by praziquantel treatment (S9C Fig). Praziquantel treatment did not affect production of other worm antigen-stimulated splenic cytokines or local mast cell protease levels in both genotypes (S9D–S9F Fig).

Serum levels of neither IgG1 nor IgG2*α* were altered in infected WT or *Pou2f3*^*-/-*^ mice at 8 dpi compared to control uninfected mice (S1 Table).

Flow cytometric immunophenotyping showed increased numbers of CD19^+^B cells, CD8^+^ T cells and CD4^+^ T cells in the mesenteric lymph nodes of both *H*. *diminuta*-infected WT and *Pou2f3*^-/-^ mice at 11 dpi compared to naïve controls (S2 Table) (S10 Fig provides the gating strategy). Immunophenotyping of the Peyer’s patches and spleen did not reveal any significant changes in number or frequency of CD19^+^B cells, CD8^+^ T cells or CD4^+^ T cells upon infection with *H*. *diminuta* when compared to control, nor when comparing between the two genotypes (S2 Table).

**Fig 8:**
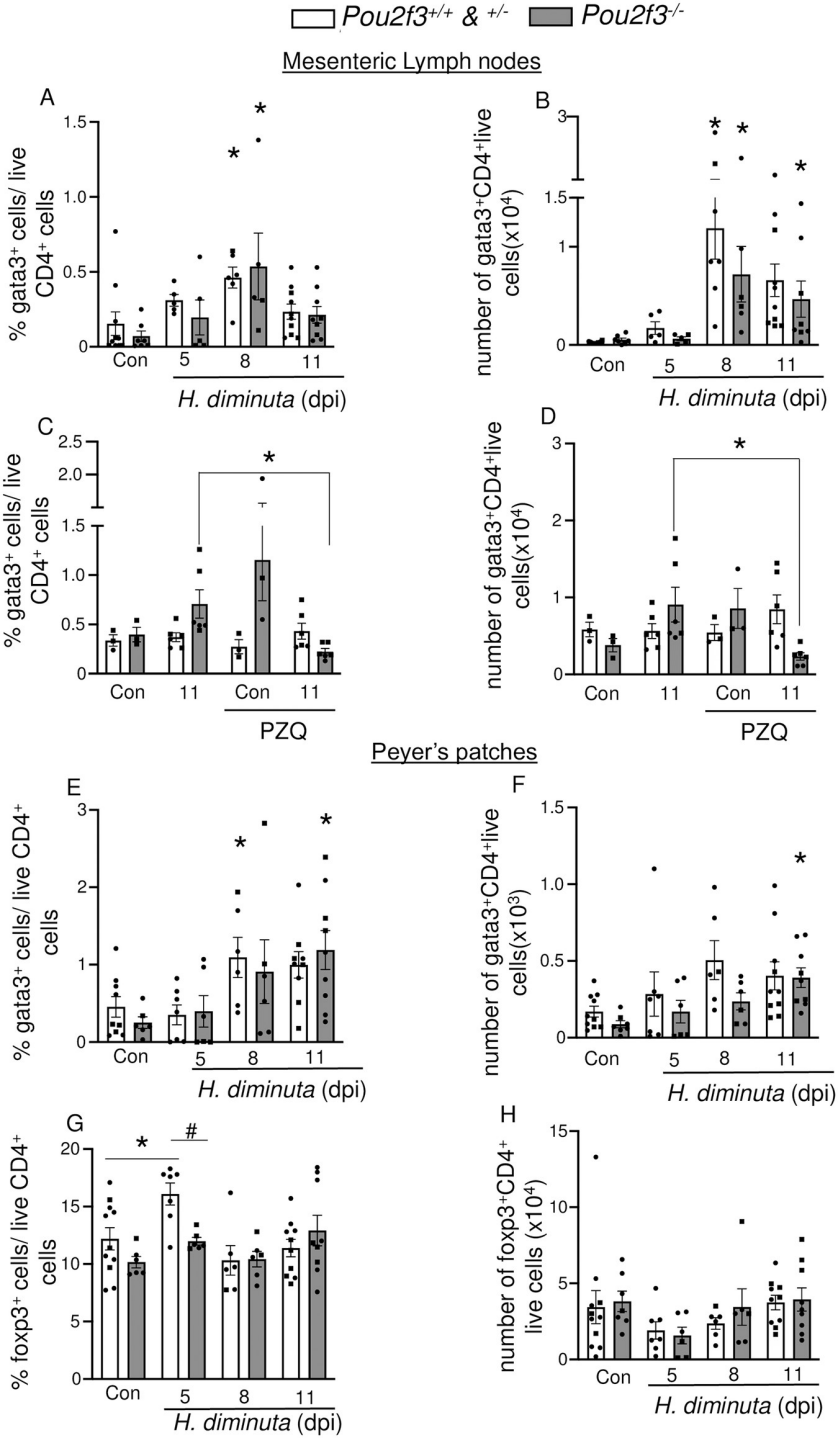
Immunophenotyping reveals subtle changes in infected wild type mice compared to infected *Pou2f3*^-/-^ mice. Male (●) and female (◼)*Pou2f3*^+/+, +/-, -/-^ mice were infected with 5 cysticercoids of *H*. *diminuta* and assessed at days post-infection (dpi). In **(C, D)**, mice were treated with praziquantel (PZQ; 1 mg/mouse by oral gavage) at 8 dpi prior to necropsy. GATA3^+^ CD4^+^ lymphocytes and Foxp3^+^ CD4^+^ lymphocyte populations were analysed by flow cytometry using single cell suspensions isolated from the **(A-D)** mesenteric lymph nodes and **(E-H)** Peyer’s patches (without ConA stimulation) and presented as **(A, C, E, G)** a percentage of total live CD4^+^ lymphocytes and **(B, D, F, H)** as cell numbers. Data are mean ± SEM, n = 3-10/group, pooled from 1–3 experiments, * p<0.05 compared to genotype matched uninfected (Con) mice analysed by two-way ANOVA followed by Dunnett’s post-test and # p<0.05 analysed by multiple *t* tests with Hom-Sidak correction comparing between genotypes.

Further investigation of the subsets of CD4^+^T cells revealed subtle changes- both infected WT and *Pou2f3*^*-/-*^ show increased number and frequency of Gata3^+^ CD4^+^ T cells in the mesenteric lymph nodes (MLN) and Peyer’s patches (Fig 8A, 8B, 8E and 8F). Infected *Pou2f3*^*-/-*^, similar to WT mice, had elevated frequencies and numbers of MLN Gata3^+^ CD4^+^ T cells at 8 dpi (Fig 8A and 8B). Helminth clearance at 8 dpi (by praziquantel treatment), reduced the frequency and number of MLN Gata3^+^ CD4^+^ T cells at 11 dpi in the *Pou2f3*^*-/-*^ mice (Fig 8C and 8D).

In Peyer’s patches, both WT and *Pou2f3*^-/-^ mice show increased Gata3^+^ CD4^+^ T cells, however this increased frequency occurred earlier in WT mice than in *Pou2f3*^*-/-*^ mice—peaking at 8 dpi rather than at 11 dpi (Fig 8E–8F). Foxp3^+^ regulatory T cells were only increased in cells isolated from Peyer’s patches of WT, but not *Pou2f3*^*-/-*^ mice at 5 dpi (Fig 8D) (intriguingly, although not pursued here, there was a statistically significant reduction in the yield of live cells from WT and *Pou2f3*^*-/-*^ mice at 5 dpi (S10 Fig)). In the MLN, T-bet^+^ T cells were increased in both WT and *Pou2f3*^-/-^mice at 11 dpi (S2 Table). There were no differences in T-bet^+^ and RORɣT^+^ T cells between the groups in any of the secondary lymphoid organs or over the course of infection (S2 Table).

### *H*. *diminuta*-induced tuft cell hyperplasia is not necessary for concomitant immunity against *Heligmosomoides bakeri*

**Fig 9:**
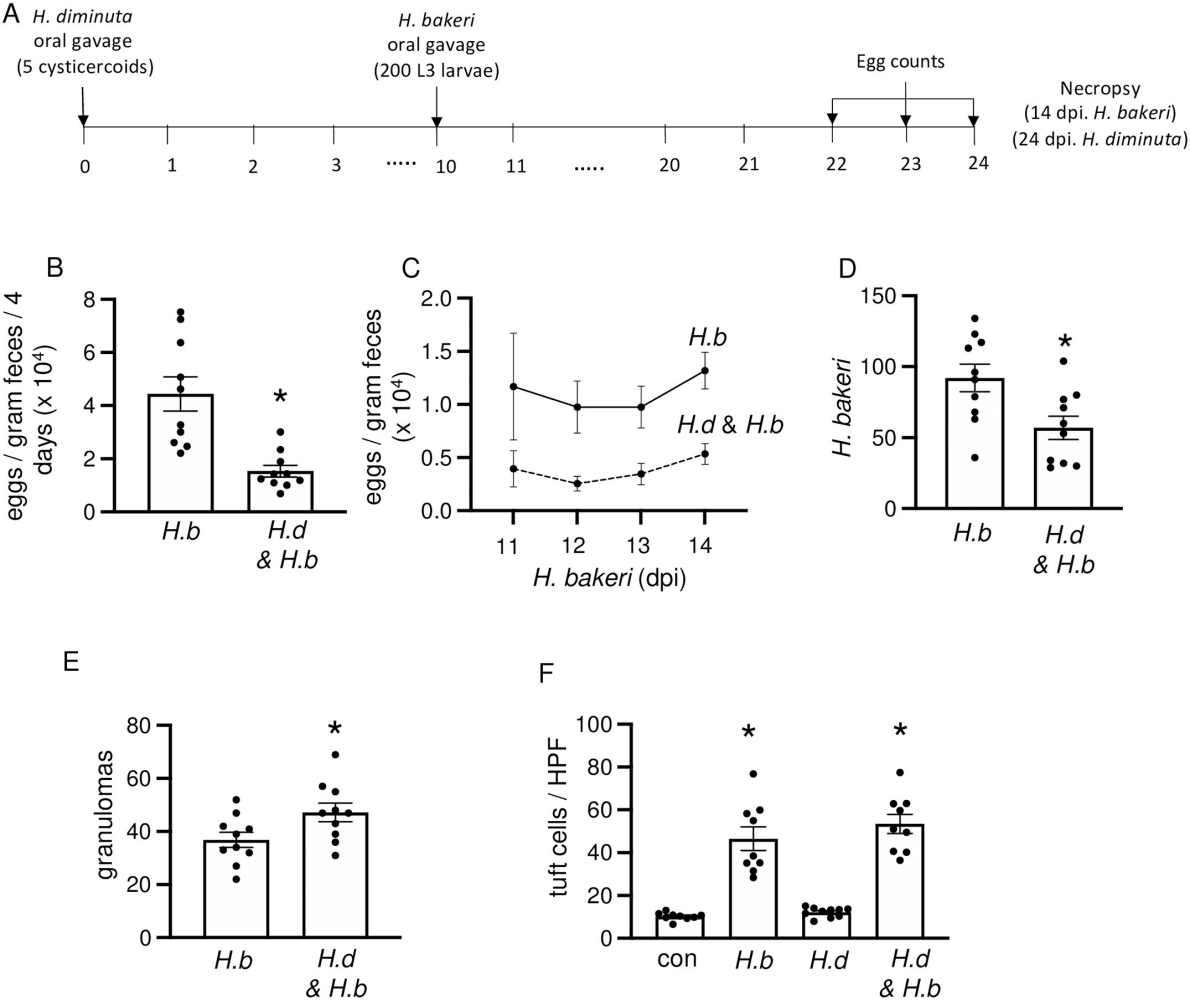
*H*. *diminuta-*infection 10 days prior to *H*. *bakeri* provides protection against the nematode. **(A)** Male C57BL/6 mice were infected with 5 cysticercoids of *H*. *diminuta* (*H*.*d*.), then 200 L3 *H*. *bakeri* (*H*.*b*.) 10 days later and necropsied at 24 days post-infection (dpi) with *H*. *diminuta*. Single parasite-infected and naïve mice served as controls. **(B-E)** In this experimental setting, prior infection with *H*. *diminuta* resulted in reduced *H*. *bakeri* egg output, fewer worms, and more granulomas. **(F)** DCLK1^+^ tuft cells were enumerated per high power field (HPF) of view (40x objective) in swiss rolls made from the first 10 cm of small intestine. Data are mean ± SEM; n = 10/group, pooled from 2 experiments, *p <0.05 compared to *H*. *bakeri* only mice or control (con) uninfected mice (**B, D, E)** by Welch’s unpaired *t*-test or **(F)** Browns Forsythe and Welch’s ANOVA test and Dunnet’s post- test for multiple comparisons.

At 11 dpi, the *H*. *diminuta*-evoked tuft cell hyperplasia occurs at a time when the worm has been rejected from the mouse, raising questions about the functional significance of the increased number of tuft cells. We postulated that tuft cell hyperplasia contributes to enhanced protection against subsequent infection with parasitic helminths. To test this, mice were infected with *H*. *diminuta* and then 10 days later with *H*. *bakeri* so that nematode larvae (L3) enter a tuft cell-enriched gut, as described in Fig 9A. In this paradigm, infection with *H*. *diminuta* resulted in an obvious protection against *H*. *bakeri*: fecal egg counts were significantly reduced, fewer adult nematodes were retrieved from the lumen and there were more jejunal granulomas in the co-infected mice (Fig 9B–9E). Consistent with published data, infection with *H*. *bakeri* resulted in DCLK1^+^ tuft cell hyperplasia [5,11,23], but the tuft cell hyperplasia was not affected by co-infection with *H*. *diminuta* (Fig 9F). Intestine from *H*. *diminuta* infected mice at 24-dpi did not show tuft cell hyperplasia at this time point.

In another experimental paradigm, mice were infected with *H*. *diminuta* and then 2 days later with *H*. *bakeri* so that the adult nematode would emerge into a gut enriched in tuft cells (S11A Fig). In this paradigm however, infection with *H*. *diminuta* had minimal impact on the *H*. *bakeri*-infection, with no statistical differences in egg output, adult *H*. *bakeri* in the lumen, or granulomas between co-infected and *H*. *bakeri*-only infected mice (S11B–S11E Fig). Consistent with the observation from (Fig 2) for BALB/c mice, intestine from *H*. *diminuta*-only infected C57BL/6 mice at 16 dpi showed no increase in jejunal tuft cells (S11F Fig).

**Fig 10:**
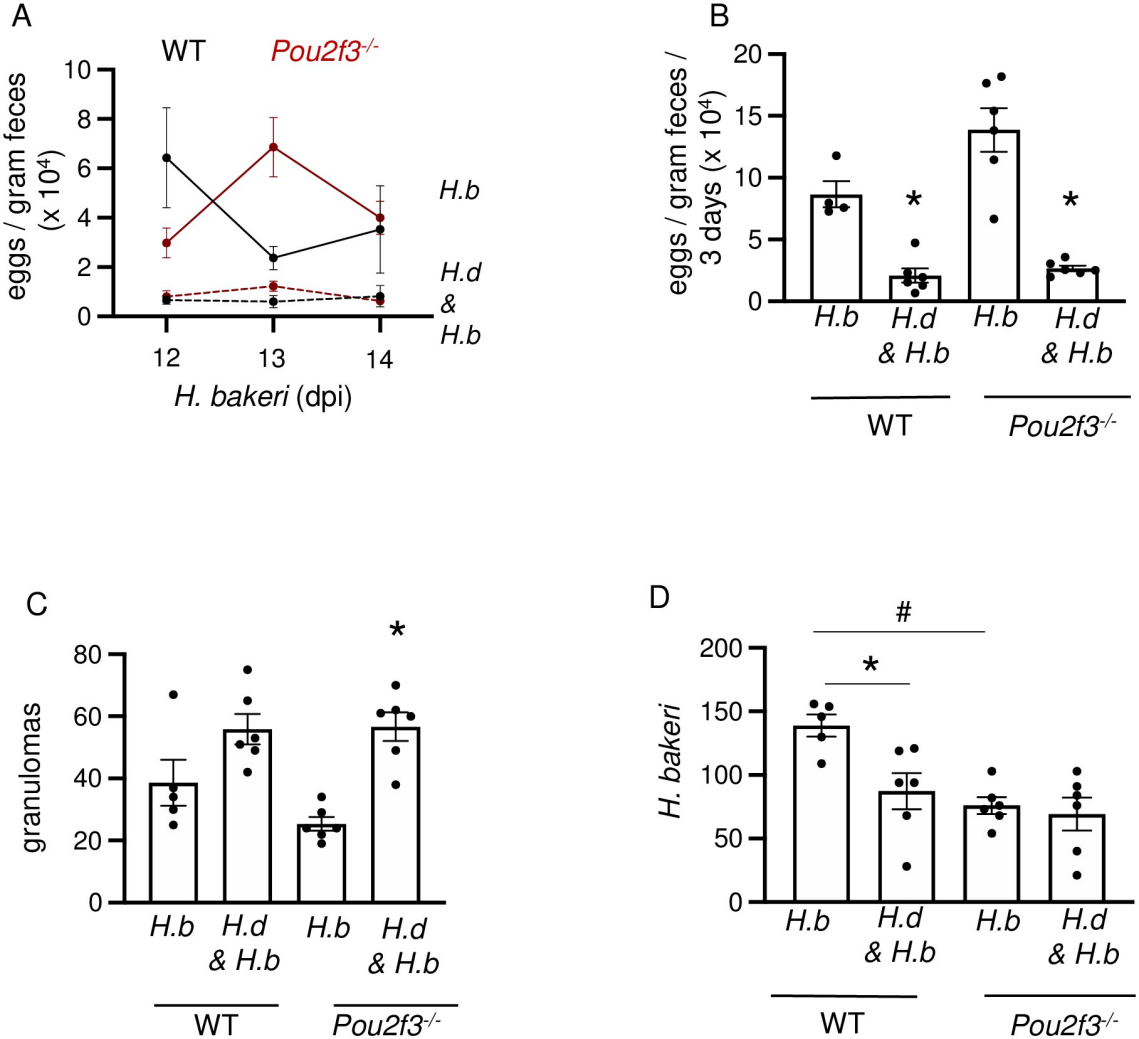
*H*. *diminuta*-induced protection against secondary infection with *H*. *bakeri* is tuft cell independent. Male C57BL/6 or *Pou2f3*^-/-^ mice were infected with 5 cysticercoids of *H*. *diminuta (H*.*d*.*)*, then 200 larvae of *H*. *bakeri (H*.*b*.*)* 10 days later and necropsied at 24 days post-infection (dpi) of *H*. *diminuta*. Single parasite infected and naïve mice served as controls. Co-infected C57BL/6 and *Pou2f3*^-/-^ mice show **(A-C)** reduced egg burden and increased numbers of granulomas. **(D)** Co-infected C57BL/6 but not *Pou2f3*^-/-^ mice show reduced adult luminal worms. Data are mean ± SEM values, n = 5-6/group, * and # represent *p* <0.05 analysed by Browns Forsythe and Welch’s ANOVA test and Dunnett’s post- test for multiple comparisons, where * compared to *H*. *bakeri* only mice of respective strain and # compared to wild-type *H*. *bakeri* only mice.

To further test if tuft cells were involved in the anti-*H*. *bakeri* effect afforded by prior infection with *H*. *diminuta* in the first paradigm tested, experiments were performed with *Pou2f3*^*-/-*^ mice. WT and *Pou2f3*^*-/-*^ mice had similar patterns of granulomas and reduced egg output (Fig 10A–10C), indicative of a lack of role for tuft cells in the cross-protection against *H*. *bakeri* after exposure to *H*. *diminuta*. Unexpectedly, lower *H*. *bakeri* worm burdens were observed in the *Pou2f3*^*-/-*^ mice compared to WT mice and worm counts in *Pou2f3*^*-/-*^ mice were not affected by co-infection with *H*. *diminuta* (Fig 10D).

## Discussion

Parasitic helminths exert a major impact on global socio-economic health and while much effort has been directed at defining anti-worm effector mechanisms [24,25], early events in the host’s recognition of a helminth are less well understood. A few ways by which hosts detect enteric helminths have been proposed, such as tissue injury, activation of antigen presenting cells, and the triggered release of alarmins from the epithelium [26–28]. The enteric tuft cell has emerged as a sentinel to signal the presence of helminths by releasing the alarmin, IL-25, to mobilize Th2-immunity [3–5,8], a paradigm based largely on studies of nematodes, especially *N*. *brasiliensis* and *H*. *polygyrus*. We show that expulsion of *H*. *diminuta* is delayed, but not negated, in the absence of tuft cells, and, intriguingly, that the kinetics of systemic Th2 immune events in the tuft cell-deficient mice were not appreciably different from *Pou2f3*^*+/+*^ WT mice.

Unlike WT C57BL/6 mice that expel *H*. *diminuta* by 8 dpi, two-thirds of *Pou2f3*^*-/-*^ mice harboured worms at 11 and 14 dpi. Similarly, *Pou2f3*^*-/-*^ mice harbour a small population of adult *N*. *brasiliensis*, for more than a month past the timepoint by which WT mice completely clear the infection [3]. The reduced worm burdens in the *N*. *brasiliensis* model [3,11] and ultimate expulsion in *H*. *diminuta* from *Pou2f3*^*-/-*^ mice highlights the value of redundancy in the detection of parasites such that the host is not left totally vulnerable should one arm of their defence system fail. The increased *Il25* mRNA observed in jejunal tissue from *H*. *diminuta*-infected mice [29] would promote local Th2 immunity [30], and the absence of *Il25* in jejunal epithelial extracts of infected *Pou2f3*^*-/-*^ mice observed here could underlie slower expulsion of the worm from the gut.

*H*. *diminuta*-infected rats and mice display shifts in the composition of their enteric microbiota [19,31]. Therefore, before assessing any immunological deficiencies underlying slowed expulsion of *H*. *diminuta* from *Pou2f3*^*-/-*^ mice, involvement of the gut microbiota was considered. Fecal bacteria compositional analyses based on 16S rRNA sequences did not reveal major differences between *Pou2f3*^*+/+*^, *Pou2f3*^*+/-*^ and *Pou2f3*^*-/-*^ littermates and a homozygous C57BL/6 *Pou2f3*^*-/-*^ colony (greater differences in the microbiota were noted between C57BL/6 *Pou2f3*^*+/+*^ mice from a commercial vendor and the in-house *Pou2f3*^*-/+*^ colony). *Pou2f3*^*-/-*^ mice from both sources displayed similar delays in the expulsion of *H*. *diminuta*, suggesting that the slowed worm expulsion from tuft cell-deficient mice is unlikely to be due to the gut microbiota; a position consistent with data showing normal temporal kinetics of expulsion of *H*. *diminuta* from germ-free mice [19]. Thus, local enteric events and systemic immunity in *Pou2f3*^*-/-*^ mice were examined for deficiencies that could account for the prolonged infection with *H*. *diminuta*.

Goblet cells, eosinophils, CD4^+^ Th2 cells, ILC2s and mast cells are some of the local cell types that contribute to expulsion of enteric helminths [32,33]. The goblet cell response that follows infection with *H*. *diminuta* [33] was delayed in *Pou2f3*^*-/-*^ mice, possibly as a consequence of reduced or delayed IL-4/IL-13 signaling downstream of tuft cell activation [3,5]. In addition, small intestinal tuft cells produce prostaglandin (PG)-D_2_ and have the cellular machinery to make acetylcholine (ACh) [6,11,34–37]; both promote mucus secretion from goblet cells [34,38] and could be involved in modulating the goblet cell response to *H*. *diminuta*. Additionally, the goblet cell mucin *Muc2* expression is reported to inversely correlate with *H*. *diminuta* biomass in rats [39] and hence the delay in goblet cell hyperplasia and concomitant *Muc2* expression at 8 dpi could influence worm fitness in *Pou2f3*^*-/-*^ mice (Fig 5C).

Increased secretion of water and mucus into the lumen coupled with increased peristalsis creates a “weep and sweep” phenomenon to propel enteric parasites caudally. While *Pou2f3*^*-/-*^ mice displayed no statistically significant changes in tonic or stimulated Isc, their small intestinal transit was significantly delayed compared to WT littermates. This would be expected to contribute to delayed worm expulsion from the tuft cell-deficient mice. Supporting this position, slowing of small intestinal transit has been described in the permissive rat host 8 dpi with *H*. *diminuta* [40,41].

Eosinophilia and mastocytosis are common reactions in response to infection with parasitic helminths, including *H*. *diminuta* [22,42,43] with the contribution of each cell type to the worm expulsion being helminth-host specific [44–47]. While the blood eosinophilia exhibited by infected *Pou2f3*^+/+^ mice was not observed in *Pou2f3*^*-/-*^ mice at 11 dpi, jejunal eosinophilia was not appreciably different at 14 dpi between both genotypes. Similarly, jejunal mast cell protease content showed no significant differences between infected mice from both genotypes. These data suggest that the delay in worm expulsion of *H*. *diminuta* may not be mediated by local deficiency in eosinophils or mast cells in the tuft cell deficient mice [48]. However, future studies should consider the activation status of these cells at earlier time points during the infection and their mediator content in infected mice of both strains.

The systemic immune response following infection with *H*. *diminuta* was similar in *Pou2f3*^*-/-*^ and WT mice. Worm-antigen stimulation of splenocytes from *H*. *diminuta*-infected *Pou2f3*^*+/+*^ and *Pou2f3*^*-/-*^ mice evoked similar amounts of Th2 cytokines indicative of immunological memory, while mitogen stimulation of splenocytes from the two strains of mice resulted in similar productions of IL-4 and IL-10. The exception here was increased IL-4 output from Con-A stimulated splenocytes from 11 dpi *Pou2f3*^*-/-*^ mice with a parallel increase in GATA3^+^ MLN cells at 8 dpi; both effects were reduced upon treatment with the anti-helminthic. This indicates that prolonged presence of *H*. *diminuta* in *Pou2f3*^*-/-*^ mice causes sustained antigenic stimulation of the immune system in these mice when compared to WT littermates.

Immunophenotyping revealed no significant differences in the frequencies of CD4^+^ T cells, CD8^+^ T cells or CD19^+^ B cells in the spleens, Peyer’s patches or mesenteric lymph nodes of *Pou2f3*^*+/+*^ and *Pou2f3*^*-/-*^ mice infected with *H*. *diminuta*: changes observed in WT mice were observed in the tuft cell-deficient mice (Fig 8 and S2 Table). However, the possibility of differential activation in any of these broad groups of lymphocytes, and a role for myeloid innate immune cells also needs to be considered. For example, neutrophils and macrophages are engaged in the murine response following infection with *N*. *brasiliensis* [49] and *in vitro* analysis has shown that an extract of *H*. *diminuta* directly affects migration of both of these cell types [50,51]. Complementing previous publications that show increased *Foxp3* mRNA expression in infected mice [29,52], Foxp3^+^ T cells were increased in the Peyer’s patches of infected *Pou2f3*^*+/+*^. *H*. *diminuta*-infected *Pou2f3*^*-/-*^ mice did not show this immunological change, and the significance of this, if any, to the slower expulsion of the worm has yet to be determined.

Mice infected with *H*. *diminuta* displayed small intestinal DCLK1^+^ tuft cell hyperplasia, and germ-free mice also developed tuft cell hyperplasia, although of slightly reduced magnitude compared to conventional C57BL/6 mice (~5-fold compared to ~8 fold at 11 dpi). Complementing these data, both WT and antibiotic-treated mice had increased numbers of jejunal tuft cells after infection with *H*. *bakeri* [53]. Thus, while bacteria-derived metabolites such as succinate can activate tuft cells [14,34,54], gut bacteria are not a prerequisite for tuft cell hyperplasia consequent to infection with *H*. *diminuta*, which is the remit of immunological signalling.

The tuft cell hyperplasia was IL-4/IL-13-dependent in BALB/c mice, and ~85% of the response was via T and/or B cells in male C57BL/6 mice as determined by analysis of *H*. *diminuta*-infected *Il-4rα*^*-/-*^ and *Rag1*^*-/-*^ mice, respectively. These data fit the paradigm in which infection with helminth parasites results in tuft cell hyperplasia driven by a feed-forward loop whereby tuft cell-derived IL-25 activates local ILC2s to release IL-13 [3,5]. To test the relevance of this paradigm in the *H*. *diminuta*-mouse model, ILC2s were partially depleted in male *Rag1*^*-/-*^ mice by CD90.2 immunoneutralization: this did not affect the mild increase in tuft cell numbers in *H*. *diminuta*-infected *Rag1*^*-/-*^ mice and may suggest another source of IL-4/IL-13 in the *Rag1*^*-/-*^ mouse. This is not unexpected as host-parasite specificity is a basic principle of parasitology; indeed, the requirement for ILC2s in host response to infection with *H*. *polygyrus* has not been established [55]. In addition, a recent study showed that a population of CD90^-^ or CD90^low^ ILC2s exist in *Rag-2*^*-/-*^ mice, that produce IL-13 and can be activated by IL-25 [56]. Given that our ILC2 depletion strategy does not affect CD90.2^-/low^ ILC2s, further testing using mouse models such as the *Rag-2*^*-/-*^*Il2rg*^*-/-*^ that lack all ILCs would be important to analyse if this subset of ILC2s contributes to the tuft cell hyperplasia observed in in *H*. *diminuta*-infected *Rag1*^*-/-*^ mice [57].

Eosinophils were increased in the gut of the anti-CD90.2 antibody treated *H*. *diminuta*-infected *Rag1*^*-/-*^ mice. Eosinophil-derived IL-4 has been identified as a driver of macrophage activation in mice following infection with the nematode *Strongyloides venezuelensis* or the protozoan *Leishmania major* [58,59]. Yet, IL-5 immunoneutralization leading to eosinophil depletion failed to abrogate the increase in jejunal tuft cells in CD90.2^+^ILC2-depleted *Rag1*^*-/-*^ mice infected with *H*. *diminuta*. Thus, the cell responsible for the residual tuft cell hyperplasia in infected *Rag1*^*-/-*^ remains to be identified. Mastocytosis characterizes the gut of *H*. *diminuta*-infected Swiss albino mice at 9 dpi [22], and mast cells can produce IL-4 [60]. NK T cells (still present in *Rag-1*^*-/-*^ mice) also produce IL-4 in response to certain parasitic infections [61]. Future studies should test the possibility that mast cell or NK cell activation in the *Rag1*^*-/-*^ mouse mediates the subtle tuft cell hyperplasia that occurs after infection with *H*. *diminuta*. Finally, IL-4/IL-13-independent, injury-induced tuft cells in murine airways [62] and DDX5-regulated intestinal tuft cells have been reported [63]. While studies in BALB/c *Il-4rα*^*-/-*^ mice indicate that the *H*. *diminuta*-evoked tuft cell hyperplasia was entirely dependent on IL-4/IL-13 signalling, the possibility of an enteric IL-4-independent tuft cell response in the gut of male C57BL/6 *Rag1*^*-/-*^ mice should not be excluded.

*H*. *diminuta*-induced tuft cell hyperplasia occurred in WT mice at a time point when worms had been effectively expelled, similar to mastocytosis following infection/expulsion of *N*. *brasiliensis* or *T*. *spiralis* in rodents [64,65]. This may reflect the consequences of mobilization of IL-4/IL-13 and the kinetics of tuft cell differentiation from the crypt stem cell [66], but also prompts questioning the biological relevance of this tuft cell-rich environment. There are many examples where helminth co-infections impact the host’s response to one or both parasites [67–69]. So, do increased numbers of tuft cells following infection with *H*. *diminuta* prime the host to detect and more rapidly respond to a second infection? To address this question, mice were infected with *H*. *diminuta* and then *H*. *bakeri* (secondary infection with *H*. *diminuta* was not used to negate issues of immunological memory [33]). In this scenario, *H*. *bakeri* L3 were introduced to the *H*. *diminuta* ‘educated’ mouse gut, there was a heightened anti-nematode response as shown by decreased worm burden and egg output (reflecting fewer worms and/or reduced fecundity) and increased numbers of granulomas. In contrast, when adult *H*. *bakeri* emerged into the lumen of a tuft cell-enriched gut due to prior infection with *H*. *diminuta* there was minimal impact on *H*. *bakeri* as assessed by the indices measured here, the most notable effect being an increased worm burden. The enhanced anti-nematode response due to priming by *H*. *diminuta* was not dependent on tuft cells because WT and *Pou2f3*^*-/-*^ showed similar responses, and so likely reflects the consequences of the cestodes’ promotion of Th2-immunity and downstream effector mechanisms [15].

In conclusion, in the absence of tuft cells, deficiencies in mucosal, not systemic, immunity, slow the expulsion of *H*. *diminuta* from mice. Infected mice display small bowel tuft cell hyperplasia that is dependent on IL-4r*α* signalling, with T and/or B cells being the major source(s) of the IL-4/IL-13: the cellular source of IL-4/IL-13 driving a 3-fold tuft cell increase in infected male *Rag1*^*-/-*^ mice awaits identification but is unlikely to be CD90.2^+^ILC2s or eosinophils. Tuft cell hyperplasia occurs at a time when *H*. *diminuta* is no longer present in the mouse, and data were not obtained to support the hypothesis that the increase in tuft cells provided increased protection against secondary infection with an unrelated helminth, which may be dependent on adaptive Th2-skewed systemic immunity after infection with *H*. *diminuta*. Thus, tuft cell responses, while important for timely clearance of worm, are neither crucial for murine recognition and overall resistance against *H*. *diminuta*, nor concomitant protection against subsequent infection with a parasitic helminth.

## Materials and methods

### Ethics

All animal experiments were approved by the Animal Care Committee of the University of Calgary (protocols AC21-0080 and AC21-0076) and performed in compliance with the guidelines established by the Canadian Council on Animal Care.

### Animals

C57BL/6 and BALB/c mice (Charles River, Montréal, QC), *Il-4rα*^*-/-*^ mice (BALB/c background), *Rag-1*^*-/-*^ mice (C57B6.129S7-Rag1<tm1Mom>/J, Jackson labs, bred at Univ. Calgary), *Pou2f3*^-/-^ mice (C57BL/6N-Pou2f3<tm1. 1(K0MP) VIcg>/Tcp; kindly provided by Dr. Jakob Von Moltke, Univ. Washington, bred at Univ. Calgary) and Sprague Dawley rats (4 months old, Charles River) were housed in specific pathogen free (SPF) conditions at the Univ. Calgary (exposed to 12h light: dark cycles, maximum of 5 mice/cage and bedding changed once weekly). Tuft cell deficient mice were bred with parents *Pou2f3*^*-/-*^ X *Pou2f3*^*-/-*^ (“Homozygous colony”) or *Pou2f3*^*-/+*^ X *Pou2f3*^*-/+*^ (obtained by backcrossing *Pou2f3*^-/-^ mice with wild type C57BL/6 mice), to yield littermates. Mice were genotyped by PCR (see S3 Table for primer sequences), performed by the Center for Genome Engineering at Univ. Calgary. Germ-free C57BL/6 mice were provided by, housed and orally gavaged in the International Microbiome Centre (IMC), Univ. Calgary. Male and female mice were used between 8–14 weeks of age and showed no obvious sex-related differences in experimental readouts; data from males and females are combined.

### Parasite models

#### *H*. *diminuta*

The *H*. *diminuta* life cycle was maintained via cyclic passage through flour beetles (*Tribolium confusum*, Canada Ant Colony, Richmond Hill, ON) for the larval cysticercoid stages and Sprague Dawley rats or *Il-4rα*^*-/-*^ mice (these mice develop an established infection) for maintenance of the adult stages. Mice and rats were orally gavaged with 5 or 10 infective cysticercoids in 100 μL of PBS, respectively. For germ-free mice, 8–10 cysticercoids were washed with an antibiotic cocktail for 2h before oral gavage as described previously [19]. Mice and rats were necropsied at different time-points post-infection and their small intestines flushed with 10 mL ice-cold PBS to retrieve and enumerate *H*. *diminuta* under a dissection microscope (Zeiss, San Diego, CA) [19].

#### Co-infection model with *H*. *bakeri*

We have referred to the species of *Heligmosmoides* used in this paper as *H*. *bakeri* because the laboratory isolates were found to be genetically dissimilar to the wild *H*. *polygyrus* [17,18]. For co-infection studies, mice were first gavaged with 5 cysticercoids of *H*. *diminuta* followed by gavage with 200 L3 larvae of *H*. *bakeri* [23] at either 2- or 10-dpi with *H*. *diminuta*. Since *Pou2f3*^*-/-*^ mice were shifted between facilities within Univ. Calgary and age-matched with WT C57BL/6 mice, beddings were exchanged to reduce variation in the microbiome [70]. Briefly, portions of soiled cage bedding from both strains were mixed and redistributed between the cages weekly for two weeks before helminth-infection. Fecal pellets were collected from the mice daily between 11–14 dpi with *H*. *bakeri*, homogenized in saturated NaCl solution and eggs counted by the McMaster method [71]. At necropsy, the intact small intestine was inspected under a dissection microscope for macroscopic granulomas and then opened longitudinally and washed in 37°C RPMI 1640 (Sigma-Aldrich, St. Louis, MO) media to free the worms from the tissue. Worms were then enumerated under a dissection microscope.

#### Praziquantel treatment

Mice, wild-type and *Pou2f3*^*-/-*^, were treated with praziquantel (1 mg/mouse, diluted in PBS; Sigma-Aldrich) [72] at 8 days post-infection with *H*. *diminuta* by oral gavage (S9A Fig). Uninfected mice treated with praziquantel served as controls.

### ILC2 and eosinophil depletion

Male *Rag-1*^*-/-*^ mice were injected intraperitoneally (ip.) with 1.8 mg/mouse of anti-CD90.2 monoclonal antibody (clone 30H12, #BE0066, BioxCell, Lebanon, NH), isotype-matched rat IgG2b (clone LTF-2, #BE0090, BioxCell), or PBS only, distributed as 250 μg/mouse on days -1, 0, 2, 4, 6, 8 and 10, and 50 μg/mouse on day 1 [73,74] for depleting CD90.2^+^ILC2s and 300 μg/mouse of anti-IL-5 antibody (clone TRFK5, #MM550C, Invitrogen, Waltham, MA) or isotype-matched rat IgG1 (clone RTK2071, #400431, BioLegend, San Diego, CA), distributed as 100 μg/mouse on days 1 and 4 and 50 μg/mouse on days 8 and 10 post infection [75] (Figs 3E and S4A). On day 0, mice were infected with *H*. *diminuta* 4.5h after the antibody administration.

### Blood smear staining

Post-necropsy, a drop of blood was collected from a tail vein puncture for a peripheral blood smear which was air dried, fixed in methanol for 30 sec and stained by Giemsa Wright staining following the manufacturer’s instructions (Sigma-Aldrich). A total of 200 immune cells were counted and identified as eosinophils (pink, granulated cytoplasm and bi-lobed nucleus), neutrophils (multi-nucleated cells with no cytoplasmic staining) or mononuclear cells in random high power field views and % eosinophils over total lymphocytes calculated [75].

### Cytokine measurement

Following a published protocol [76], spleens were chopped into ~1–2 mm^2^ pieces, forced through a 100 μm nylon mesh (Fisherbrand, ThermoFisher Scientific, Waltham, MA) and treated with ammonium-chloride-potassium (ACK) solution for 1.5 min to lyse red blood cells. The lymphocyte yield was resuspended at 5x10^6^ cell/mL in RPMI buffer 1640 (Sigma-Aldrich) with 10% (vol./vol.) fetal bovine serum (FBS) (Avantor, Radnor, PA). Lymphocytes were stimulated with the mitogen, concanavalin A (2 μg/mL; Sigma-Aldrich) for 48h or a PBS-soluble crude extract of adult *H*. *diminuta* (HdAg, 200 μg/mL) [77] for 96h, supernatants were collected, and cytokines measured by ELISA according to manufacturer’s instructions (Duo-set ELISA (IL-4,-IL-5 and IL-10), R & D Systems, Minneapolis, MN or Mouse IL-13 uncoated ELISA, Invitrogen). Readings below the bottom standard on the curve were considered as below level of detection/zero.

### Tissue mast cell protease (MCPT-1) quantification

At necropsy, ~1 cm portions of the mid-jejunum were isolated, snap frozen and later homogenized using the Polytron PT-MR1200 homogenizer in 1 mL ice-cold PBS with protease inhibitor cocktail (Complete, Roche diagnostics, Indianapolis, IN) and 0.1% Tween-20 (Sigma- Aldrich). Samples (diluted to a total protein concentration of 0.5 mg/mL assessed by Bradford assay (BioRad laboratories Canada, Mississauga, ON)) were analysed for mast cell protease-1 concentration by ELISA according to manufacturer’s instructions (Invitrogen) [48].

### Histology and immunohistochemistry

Segments (~1 cm) of mid-jejunum, mid-ileum, proximal and distal colon were preserved in Zamboni’s fixative (4% paraformaldehyde, 12.5% picric acid in 0.1 M phosphate buffer, pH 7.3) for 24h, transferred to a 20% sucrose/PBS solution for 12-16h and cryopreserved in optimal cutting temperature compound (OCT, Tissue-Tek, Sakura Finetek, Torrance, CA). Adjacent tissue segments were fixed in 10% neutral buffered formalin (VWR) for 24h and stored in 70% ethanol or fixed in methacarn fixative (60% absolute methanol, 30% chloroform, 10% glacial acetic acid) for 24h to preserve mucus in subsequent goblet cell staining [78] before dehydration and clearing in xylene (VWR) and embedding in paraffin wax (HistoplastIM, ThermoFisher Scientific).

Paraffin sections (5 μm) were de-paraffinized in xylene, rehydrated and heated in antigen retrieval buffer (10 mM monosodium citrate, pH 6.3) for 20 min at 95–100 °C. Cryosections (10 μm) were washed in 1x PBS to remove OCT prior to immunostaining. For tuft cell staining, sections were incubated with primary antibody against DCLK1 (Abcam, Cambridge, UK; 1:2,000 dilution (see S4 Table for antibody codes)) diluted in 0.1% BSA (VWR), 0.01% Triton X-100 in PBS for 16h at 4°C, washed 3 times in PBS (1x) with 0.01% Triton-X 100 (Sigma-Aldrich) for 15 min each and incubated with secondary antibodies: donkey-anti-rabbit CY3 (Jackson ImmunoResearch, West grove, PA; 1:500 dilution) for 2h at room temperature, counterstained with DAPI (100 pg/mL, 5 min, ThermoFisher Scientific), and washed 3 times in PBS for 10 min each time, before mounting with Prolong anti-fade mounting reagent (Invitrogen) and applying coverslips. For eosinophil identification, cryosections were blocked with 5% Normal goat serum (VWR) for 1h and subsequently incubated with anti-SiglecF (BD Biosciences; 1:200) and anti-IL-5R*α* antibody (ThermoFisher Scientific; 1:100) reconstituted in blocking solution for 16h at 4°C, washed as described previously and incubated with secondary antibodies: donkey anti-goat CY3 and donkey anti-rat CY5 (Jackson ImmunoResearch; 1:500 dilution) (see S4 Table for antibody codes). Images were captured from coded slides on an epi-fluorescent microscope (BX41, Olympus, Shinjuku city, Tokyo) and camera (DP40, Olympus) using the CellSense software and Image J (FIJI) for adjusting brightness of images and merging channels.

Goblet cells were identified by periodic acid Schiff staining (PAS) (Schiff’s reagent, Sigma-Aldrich) on paraffin sections as previously described [33]. Tissue eosinophils were identified by hematoxylin and eosin staining as cells with bilobed nuclei and pink cytoplasmic granules or as SiglecF^+^ cells (as above). Sections were coded and scored for tuft cell and goblet cell number per villus crypt unit (VCU; defined by an intact crypt and round villus tip (small intestine)), crypt unit (colon), or per high power field (HPF (i.e., x40 objective)) over 5 fields of view per tissue section selected using the DAPI channel or at random. QuPath software was used to identify and enumerate SiglecF^+^ cells excluded from the villus epithelium/HPF view in images taken at 40X objective over 2–3 fields of view per mouse [79].

### Epithelial and immune cell isolations

Epithelial Cells. The small intestine was excised and flushed with ice-cold PBS. Epithelial cells were isolated from the entire small intestine [80] whereas only the jejunum (i.e. 10 cm of tissue beginning 5 cm from the stomach) was use to retrieve LPLs. After washing twice with ice-cold D-PBS (Dulbelco’s PBS without Ca^2+^ and Mg^2+^, Sigma-Aldrich), and removal of fat, the intestine was opened along the mesenteric border and incubated in ice-cold D-PBS containing 30 mM EDTA (ethylenediaminetetraacetic acid, Sigma-Aldrich), 0.5 mM DTT (1,4-dithiothreitol, Sigma-Aldrich) and Rho-associated kinase (ROCK) inhibitor Y276302 (10 μM, PeproTech, ThermoFisher Scientific) for 20 min, followed by a 20 min incubation at 37°C in D-PBS, 30 mM EDTA and 10 μM Y276302. Intestinal fragments were vigorously shaken and vortexed for 30 sec (3 times), the supernatant collected, centrifuged (10 min, 300x*g*) and the pelleted material used as the epithelial-enriched fraction, which was preserved in Ribozol (VWR) for further analysis.

Lamina propria lymphocytes. Jejunal segments were cut into 2–5 mm pieces and shaken at 37°C at 250 rpm on an orbital shaker (Forma Orbital Shaker, ThermoFisher Scientific) for 20 min in HBSS (Hank’s balanced salt solution, Sigma-Aldrich) with 5% FBS and 5 mM EDTA to remove epithelial cells. Tissue was then minced and digested with collagenase (type VIII; 1 mg/mL, Sigma-Aldrich) and DNAse (1 mg/mL, New England Biolabs, Ipswich, MA) in HBSS + 5% FBS for 10 min on an orbital shaker (250 rpm, 37°C), strained through a 100 μm filter and pelleted by centrifugation at 300x*g* (10 min at 4°C).

Peyer’s patch (PP) cells. PPs along the entire small intestine were surgically excised and incubated in RPMI 1640 + 10% FBS pre-warmed at 37°C in an orbital shaker at 140 rpm for 10 min, followed by gentle disruption using a 10 mL syringe plunger pressing through a 40 μm cell strainer and then rinsed with RPMI 1640 + 10% FBS. The cell suspension was rinsed with RPMI 1640 + 10% FBS and pelleted to obtain a PP lymphocyte single cell preparation.

Mesenteric lymph node cells. MLNs were collected in DMEM + 10% FBS, disrupted by filtering through a 100 μm cell strainer to generate single cell suspension and treated with ACK lysis buffer to eliminate red blood cells. The cells will be pelleted at 350xg for 5 min at 4°C and resuspended in 500 μL of ice-cold DMEM (Sigma-Aldrich) + 10% FBS medium.

### Flow cytometry immune cell isolations

Cells were washed twice with PBS and stained with fixable viability stain (1:1,000, 15 min in the dark on ice) before being blocked with anti-mouse CD16/32 Fc blocker (BioLegend, 5 min) and then incubated with antibodies against surface antigens (S4 Table; gating strategy shown in S10A Fig) for 30 min in the dark on ice. The cells were then washed twice in permeabilization buffer (Foxp3 fix/perm buffer kit, BD Biosciences), and fixed for 20 min in Fix/Perm buffer before being stained with antibodies against intracellular transcription factors Foxp3, GATA3, RORɣT, and T-bet (S4 Table) for 30 min in the dark on ice, washed and resuspended in FACs buffer (D-PBS, 2% FBS, 0.5 mM EDTA).

### RT-PCR

Sections of mid-jejunum (~1 cm) isolated 10 cm from the stomach or isolated epithelial cells were collected in Ribozol (VWR), snap frozen and stored at -80°C until processing. Tissue segments or isolated epithelium were homogenized using a Polytron PT-MR1200 homogenizer, and the aqueous phase extracted with chloroform. Two μg RNA (measured using Nanodrop, ThermoFisher Scientific) was converted to cDNA using the iScript cDNA conversion kit (Bio-Rad) and analysed by real time PCR (RT- PCR) using iQSYBER Green Supermix (Bio-Rad) and the primers listed in S3 Table (Life Technologies, Thermo Fisher Scientific) designed using NCBI Primer-BLAST software [81]. Primers were used at 0.2 μM concentration, initial denaturing was at 95°C for 10 min followed by 40 cycles of denaturing at 95°C (15 sec) and primer annealing/extension at 60°C (1 min). Gene expression was calculated using ΔCT2 analysis and normalized to the 18S rRNA housekeeping gene within samples and to uninfected controls [82].

### Analysis of intestinal ion transport and intestinal transit

Intestinal ion transport was assessed in freshly isolated mid-jejunum mounted in Ussing chambers as previously described [83]. Following a 15 min stabilization period, baseline short circuit current (Isc, μA/cm^2^) was recorded, followed by stimulated Isc responses evoked by the cholinergic muscarinic agonist, carbachol (100 μM), and 15 min after Isc had returned to baseline, the addition of the adenylate cyclase activator, forskolin (10μM). For both secretagogues, the peak increase in Isc within 5 min of treatment was recorded.

Small intestinal transit was assessed by measuring the distance travelled by Evan’s blue dye (Sigma-Aldrich) (5% in a 5% gum arabic solution, 200 μL) 15 min post-gavage in comparison to the total length of the small intestine [84].

Colonic motility was assessed by recording the time taken for mice (anesthetised with isoflurane) to expel a 3 mm glass bead inserted 2 cm past the anus using a thin, sterile plastic catheter tube [84].

### 16S rRNA analysis of fecal microbiome

Fecal pellets (100 mg) were collected from male and female wild-type C57BL/6 mice (purchased from Charles River), homozygous *Pou2f3*^*-*/-^ mice (bred at Univ. Calgary) and littermate offspring from the *Pou2f3*^-/+^ X *Pou2f3*^-/+^ colony (bred at Univ. Calgary). Genomic DNA was extracted using DNeasy PowerSoil Pro Kit (Qiagen, Germantown, MD) and libraries prepared with two steps PCR amplification protocol targeting the V3-V4 variable region of the 16S rRNA gene. The pooled library was sequenced on Illumina MiiSeq 600 cycle kit (300 base pairs paired end) and analysis done on R studio (R version 4.3.1) as previously described [19]. Adapter and primer sequences were removed using Cutadapt program, paired end fastq files were run through the dada2 pipeline to generate an amplicon sequence variant table, and taxonomic classifications were assigned using the Silva 138.1 database. Community analysis was performed using Phyloseq version 1.44.0, α diversity was determined by “plot_richness” function and Wilcoxin rank sum test for statistical analysis, β diversity- weighted unifrac distances were plotted using principal coordinate analysis (PCoA) followed by a PERMANOVA to test for statistically significant compositional differences between groups.

### Data presentation and analysis

Data are represented as mean ± SEM unless specified and statistical analyses performed with GraphPad Prism (v. 8.2.1). Tests of normality and lognormality (Shapiro Wilk test) were employed when n>3 and assessment for outlier data points was by ROUT’s method. Parametric data were analysed by Welch’s *t* test for two groups or Brown’s Forsythe ANOVA followed by Dunnet’s post-test for multiple comparisons. Non-parametric data were analysed by Mann Whitney *t* test for two groups or Kruskal Wallis test, with Dunn’s test post-hoc analysis. When there were two variables (e.g., genotype and day post infection), data was analysed by Two-Way ANOVA and Dunnett’s/ Tukey’s test for multiple comparisons. *p* <0.05 was accepted as statistically significant difference.

## Supporting information

S1 Fig *H*. *diminuta*-infection induces murine ileal but not colonic tuft cell hyperplasia. Male BALB/c and C57BL/6 mice were infected with 5 cysticercoids of *H*. *diminuta* and assessed at days post-infection (dpi). **(A)** Representative image of a H&E-stained formalin-fixed paraffin embedded section (5 μm) of small intestinal swiss rolls prepared from C57BL/6 mice at 5 dpi revealing a longitudinal cross-section of a *H*. *diminuta* worm. DCLK-1^+^ cells were enumerated in **(B)** ileal and **(C-E)** proximal and distal colonic cryosections (10 μm), immunostained for DCLK-1 and counterstained for DAPI, per villus crypt unit (VCU) for ileum and per crypt unit (CU) for colonic sections and averaged over 20 respective units per mouse. **(C)** Representative images of proximal and distal colonic cryosections from control and infected BALB/c mice (11 dpi) immunostained for DCLK1 (red) and counterstained with DAPI (blue). On images, “L”–lumen, “V”- villus,” C”- crypt, “LP”- lamina propria and “S”- serosa. Data are mean ± SEM values, n = 4-9/group, pooled from 2–3 experiments, * *p*<0.05 compared to the control (Con) group, analysed by **(B, D)** Browns Forsythe and Welch’s ANOVA and Dunnett’s test or **(E)** Unpaired *t* test with Welch’s correction, compared to control (Con) group.
(PDF)

S2 Fig Sprague Dawley rats chronically infected with *H*. *diminuta* show tuft cell hyperplasia. Male Sprague Dawley rats were infected with 10 cysticercoids of *H*. *diminuta* and assessed 3–6 months post infection. Rodent small intestine was flushed with ice cold PBS to **(A)** enumerate scolexes of *H*. *diminuta*, **(B)** photograph, and **(C)** collect wet weights as evidence of chronic infection. **(B)** Representative image of adult *H*. *diminuta* worms collected from a rat infected for 6 months. **(D)** Mid-jejunal cryosections (10μm) were immuno-stained with anti-DCLK1 antibody and counterstained with DAPI. DCLK1^+^ cells were enumerated per high power field view (HPF) at 40X objective and averaged over 5 fields of view. Data are mean ± SEM, n = 5/group, pooled from 2 experiments, * *p*<0.05 compared to uninfected animals analysed by Welch’s *t* test.
(PDF)

S3 Fig Anti-CD90.2 treatment delivered intra-peritoneally results in depletion of CD90.2^+^ ILC populations in male *Rag-1*^*-/-*^ mice infected with *H*. *diminuta*. **(A)** Upon necropsy, cell populations from the jejunal lamina propria were inspected for Gata-3 and CD90.2 expression by flow cytometry, pre-gated as lineage^-^ live lymphocytes. **(B)** Anti-CD90.2 treatment significantly depleted the number of CD90.2^+^ Gata-3^+^cells compared to isotype/PBS controls, but did not deplete **(C)** a population of CD90.2^-^ Gata-3^+^ cells. Data are mean ± SEM, n = 6/group, pooled from 2 experiments, * *p*<0.05 analysed by Brown’s Forsyth ANOVA with Dunnett’s test compared to control (Con).
(PDF)

S4 Fig CD90.2^+^ ILC-depleted *Rag-1*^*-/-*^ mice infected with *H*. *diminuta (Hd)* have more gut eosinophils than control mice. Male *Rag-1*^*-/-*^ mice (with and without anti-CD90.2 treatment), were infected with 5 cysticercoids of *H*. *diminuta* and assessed at 11 days post infection (dpi). **(A)** Representative images of mid-jejunal cryosections stained with anti-SiglecF antibody, anti-IL-5Rα antibody and DAPI. “T” stands for tuft cells; white arrows point towards eosinophils **(B)** Eosinophils enumerated using QuPath for SiglecF^+^ cell detection over 2–3 high power fields of view (HPF) photographs taken at 40x objective. Data are mean ± SEM, n = 4-5/group, pooled from 3 experiments, * *p*<0.05 compared to control mice analysed by Brown-Forsythe ANOVA with Dunnett’s post-test for multiple comparison.
(PDF)

S5 Fig ILC2 and eosinophil depleted *Rag-1*^*-/-*^ mice infected with *H*. *diminuta (Hd)* still display tuft cell hyperplasia. **(A)** Male *Rag-1*^*-/-*^ mice (± anti-CD90.2 and anti-IL-5 antibody treatment) were infected with 5 cysticercoids of *H*. *diminuta* and assessed at 11 days post infection (dpi). **(B)** Blood eosinophil percentage analysed on Giemsa-stained peripheral tail vein blood smears show marked reduction in the anti-IL-5 treated group. **(C)** Small intestines were flushed with ice-cold PBS and contents observed under a dissection microscope for worms. **(D)** Tuft cells enumerated per VCU (Villus Crypt Unit). Data are mean ± SEM, n = 3-4/group, * *p*<0.05 compared to controls (Con), analysed by **(B)** Kruskal Wallis test with Dunn’s post-test or **(D)** Brown-Forsythe and Welch’s ANOVA with Dunnett’s post-test for multiple comparisons.
(PDF)

S6 Fig Fecal microbiome varies between mice bred in a commercial facility vs the University of Calgary facility (in-house) regardless of genotype. Fecal 16S rRNA sequencing was conducted on samples from female (●) and male (▲) C57BL/6 mice commercially purchased from Charles River (CR), homozygous *Pou2f3*^-/-^ mice bred in-house from *Pou2f3*^-/-^ X *Pou2f3*^-/-^ parents and littermates (*Pou2f3* ^-/-, -/+^ and ^+/+^) bred from *Pou2f3*^-/+^/*Pou2f3*^-/-^ X *Pou2f3*^-/+^ parents. **(A)** α diversity plots reveal significant differences in bacterial compositions of mice purchased from Charles River in the Observed and Chao1 measures of richness compared to in-house colonies, but a similarity between the in-house breeding colonies in all measures. Data are box and whisker plots: horizontal line at median, box plots show 25–75% quartiles and vertical line,—minimum and maximum value, n = 9–11, * p<0.05 analysed by Kruskal Wallis test with Dunn’s multiple comparison test compared to *Pou2f3*^-/-^ mice. **(B)** β diversity (PCoA, Weighted unifrac distance) shows separated clustering of colonies of commercially purchased mice (grouped to the right, in red) and colonies bred in-house (grouped to the left, in blue).
(PDF)

S7 Fig Bacterial relative abundance at phylum level in fecal samples. Fecal 16S rRNA sequencing was conducted on samples from female and male C57BL/6 mice commercially purchased from Charles River (CR), homozygous *Pou2f3*^-/-^ mice from *Pou2f3*^-/-^ X *Pou2f3*^-/-^ parents and littermates (*Pou2f3* ^-/-^ or ^-/+^ and ^+/+^) bred from Pou2f3^-/+/-/-^ X Pou2f3^-/+^ parents in-house.
(PDF)

S8 Fig Tuft cell deficient mice display significant Th2 systemic immune response against *H*. *diminuta*. Male (●) and female (■) *Pou2f3*^-/-^ mice were infected with 5 cysticercoids of *H*. *diminuta* and assessed at days post-infection (dpi). Cytokine ELISAs for IL-4, -10, and -13 were performed on supernatants from splenic cells (5x10^6^) stimulated with concanavalin A (2 μg/mL) for 48h. Data are mean ± SEM, n = 5-10/group, pooled from 2–3 experiments, * *p*<0.05 compared to uninfected mice (Con) analysed by Kruskal Wallis Test and Dunn’s post-test.
(PDF)

S9 Fig Praziquantel (PZQ)- induced removal of *H*. *diminuta (H*.*d)* at 8 dpi does not abrogate all systemic and local immune responses in *Pou2f3*^-/-^ mice. **(A)** Male (●) and female (■) *Pou2f3*^-/-^ and C57BL/6 mice were infected with 5 cysticercoids of *H*. *diminuta*, treated with PZQ (1 mg/mouse by oral gavage) at 8 days post-infection (dpi) and assessed at 11 dpi. **(B)** Small intestines were flushed with ice-cold PBS and contents observed under a dissection microscope for worms. **(C)** Blood eosinophil percentage was analysed on Giemsa-stained peripheral tail vein blood smears. **(D, E)** Cytokine ELISAs for IL-13 and IL-10 were performed on supernatants from splenic cells (5x10^6^) stimulated with a PBS-soluble crude extract of adult *H*. *diminuta* (HdAg, 200 μg/mL, 96h). **(F)** Mast cell protease-1 concentrations were measured by ELISA in mid-jejunal homogenates.
(PDF)

S10 Fig (A) Gating strategy and (B) total live cell yield for immunophenotyping of *Pou2f3*^+/-^ and *Pou2f3*^-/-^ mice at various days post infection (dpi). Male (●) and female (■) littermate *Pou2f3*^+/+, +/-, -/-^ mice were infected with 5 cysticercoids of *H*. *diminuta* and assessed at days post-infection (dpi). Live lymphocyte populations were analysed by flow cytometry using single cell suspensions isolated from the **(B)** mesenteric lymph nodes (MLN), **(C)** Peyer’s patches (without ConA stimulation) and **(D)** spleen. Data are mean ± SEM, n = 5-12/group, pooled from 2–3 experiments, * *p*<0.05 compared to uninfected mice (Con) of each genotype, analysed by Kruskal Wallis test and Dunn’s post-test.
(PDF)

S11 Fig Infection with *H*. *diminuta* two days prior to *H*. *bakeri* does not provide enhanced protection against the nematode. **(A)** Male C57BL/6 mice were infected with 5 cysticercoids of *H*. *diminuta* (*H*. *d*.), then 200 L3 of *H*. *bakeri (H*.*b*.*)* and necropsied at 16 days post-infection with *H*. *diminuta*. Single parasite-infected and naïve mice served as controls. **(B-E)** Co-infected mice showed no significant differences in *H*. *bakeri* egg output, luminal worms, or granulomas. **(F)** DCLK1^+^ tuft cells were enumerated per high power field (HPF) of view (40x objective) in swiss rolls made from the first 10 cm of small intestine. Data are as mean ± SEM, n = 5/group, * *p* <0.05 compared to *H*. *bakeri* only mice or control (con) uninfected mice by **(A-E)** Welch’s unpaired *t* test or **(F)** Browns Forsythe and Welch’s ANOVA test and Dunnet’s post-test for multiple comparisons.
(PDF)

S1 Table Comparison of uninfected control and *H*. *diminuta*-infected (8 dpi.) wild-type and *Pou2f3*^-/-^ littermates. (PDF)

S2 Table Immunophenotyping of uninfected *and H*. *diminuta*-infected wild-type (WT; consists of *Pou2f3*^*+/+*^ and *Pou2f3*^*+/-*^ mice) and tuft cell-deficient *Pou2f3*^*-/-*^ C57BL/6 mice. (PDF)

S3 Table Primers used for genotyping or RT-PCR on murine cells. (PDF)

S4 Table Antibodies used for flow cytometry and immunohistochemistry. (PDF)

S1 Data Excel spreadsheet containing the underlying numerical data and statistical analysis for all figures and tables. (XLSX)
