# Supplementary figures and images for: Enteric tuft cells coordinate timely expulsion of the tapeworm Hymenolepis diminuta from the murine host by coordinating local but not systemic immunity
Source: PLoS Pathog. 2024 Jul 31;20(7):e1012381. doi: 10.1371/journal.ppat.1012381 (PMC11290655; doi:10.1371/journal.ppat.1012381)

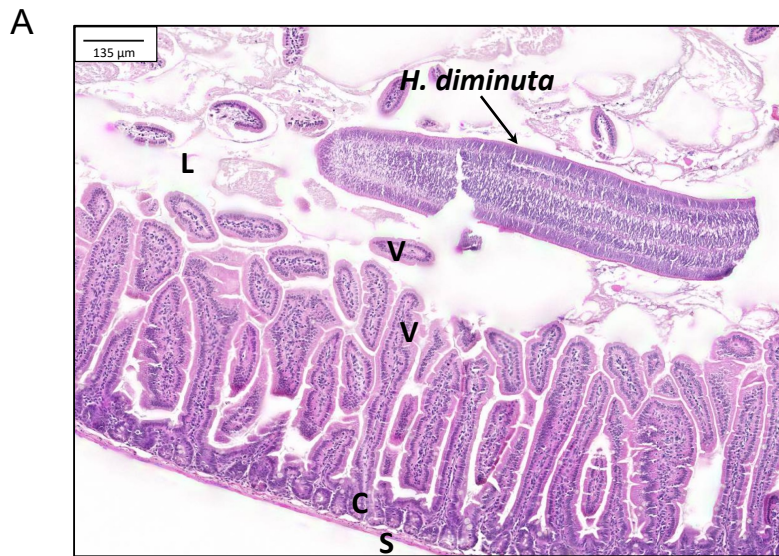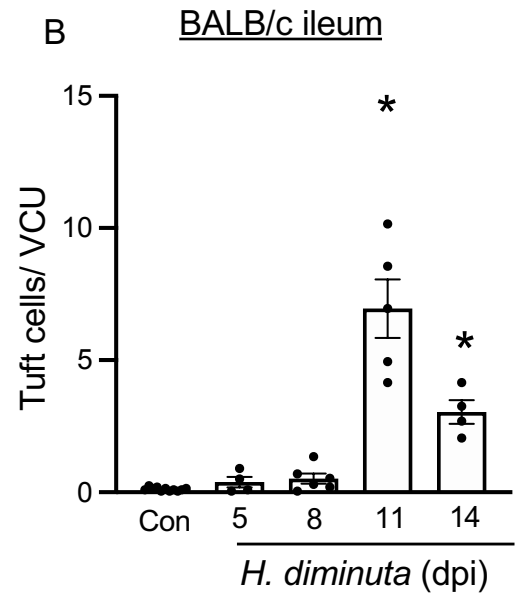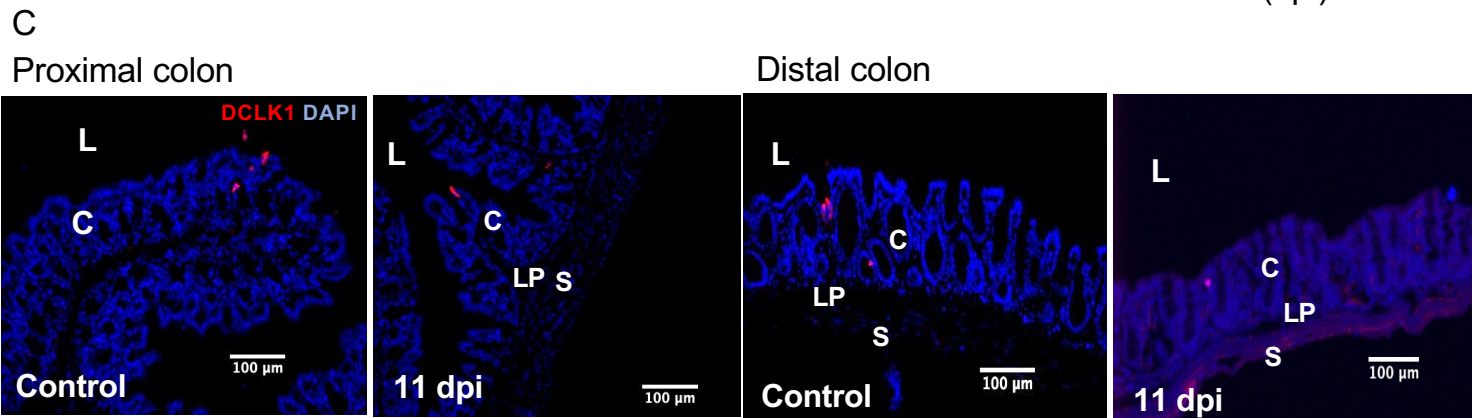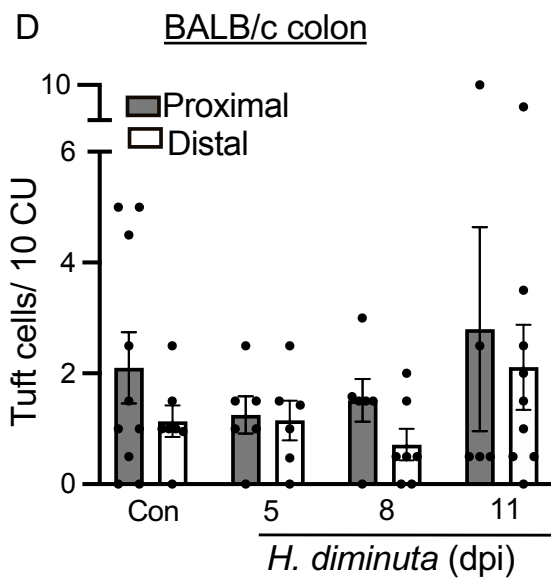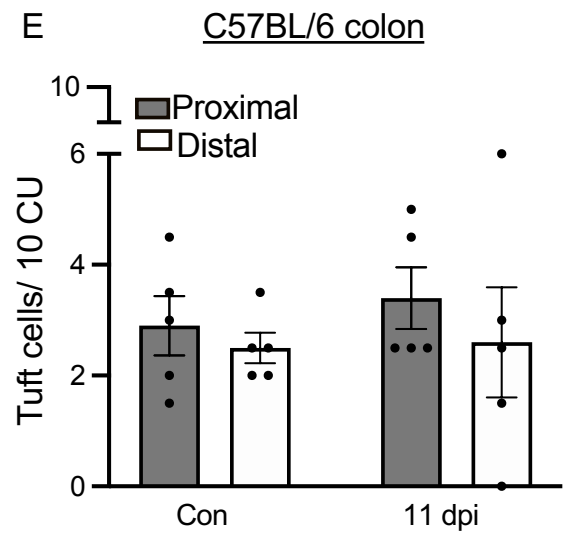

Supplement: S1 Fig — Male BALB/c and C57BL/6 mice were infected with 5 cysticercoids of H. diminuta and assessed at days post-infection (dpi). (A) Representative image of a H&E-stained formalin-fixed paraffin embedded section (5 μm) of small intestinal swiss rolls prepared from C57BL/6 mice at 5 dpi revealing a longitudinal cross-section of a H. diminuta worm. DCLK-1+ cells were enumerated in (B) ileal and (C-E) proximal and distal colonic cryosections (10 μm), immunostained for DCLK-1 and counterstained for DAPI, per villus crypt unit (VCU) for ileum and per crypt unit (CU) for colonic sections and averaged over 20 respective units per mouse. (C) Representative images of proximal and distal colonic cryosections from control and infected BALB/c mice (11 dpi) immunostained for DCLK1 (red) and counterstained with DAPI (blue). On images, “L”–lumen, “V”- villus,” C”- crypt, “LP”- lamina propria and “S”- serosa. Data are mean ± SEM values, n = 4-9/group, pooled from 2–3 experiments, * p<0.05 compared to the control (Con) group, analysed by (B, D) Browns Forsythe and Welch’s ANOVA and Dunnett’s test or (E) Unpaired t test with Welch’s correction, compared to control (Con) group. (PDF) [file ppat.1012381.s001.pdf]

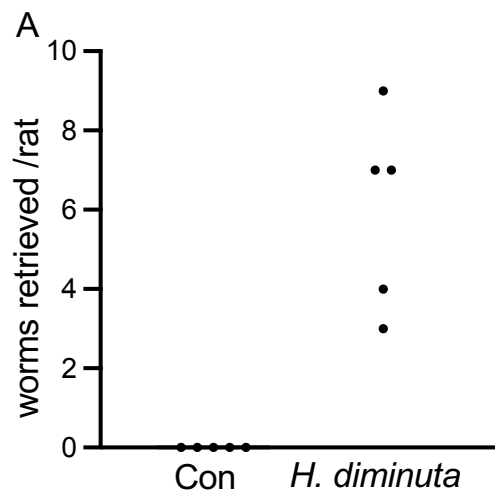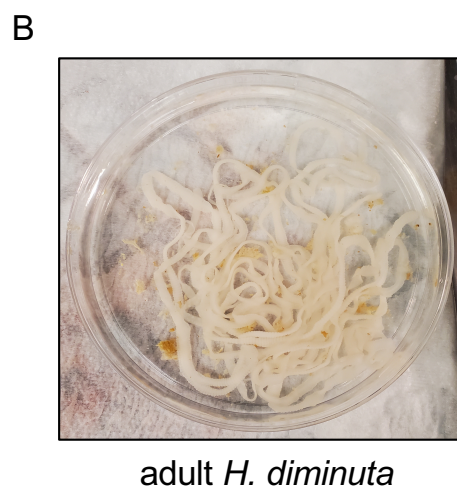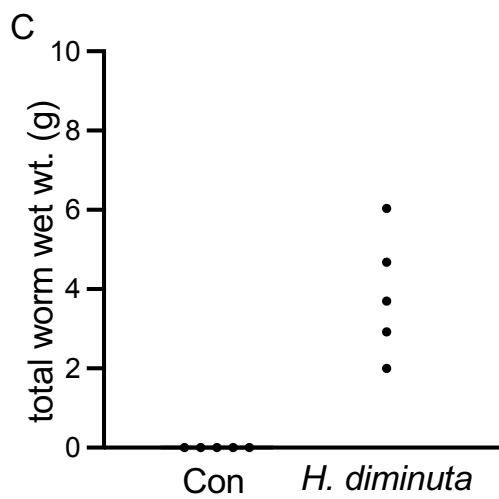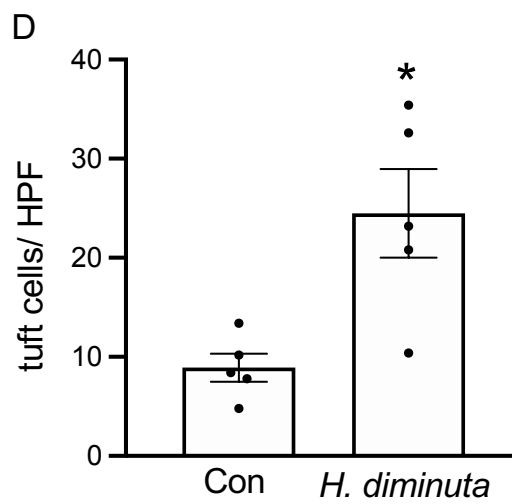

Supplement: S2 Fig — Male Sprague Dawley rats were infected with 10 cysticercoids of H. diminuta and assessed 3–6 months post infection. Rodent small intestine was flushed with ice cold PBS to (A) enumerate scolexes of H. diminuta, (B) photograph, and (C) collect wet weights as evidence of chronic infection. (B) Representative image of adult H. diminuta worms collected from a rat infected for 6 months. (D) Mid-jejunal cryosections (10μm) were immuno-stained with anti-DCLK1 antibody and counterstained with DAPI. DCLK1+ cells were enumerated per high power field view (HPF) at 40X objective and averaged over 5 fields of view. Data are mean ± SEM, n = 5/group, pooled from 2 experiments, * p<0.05 compared to uninfected animals analysed by Welch’s t test. (PDF) [file ppat.1012381.s002.pdf]

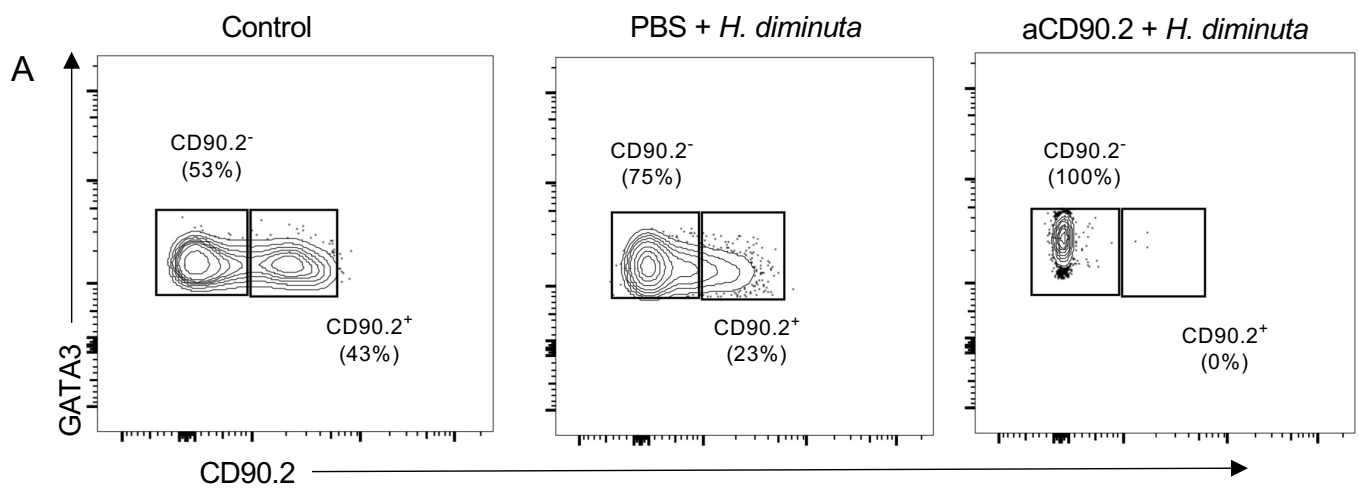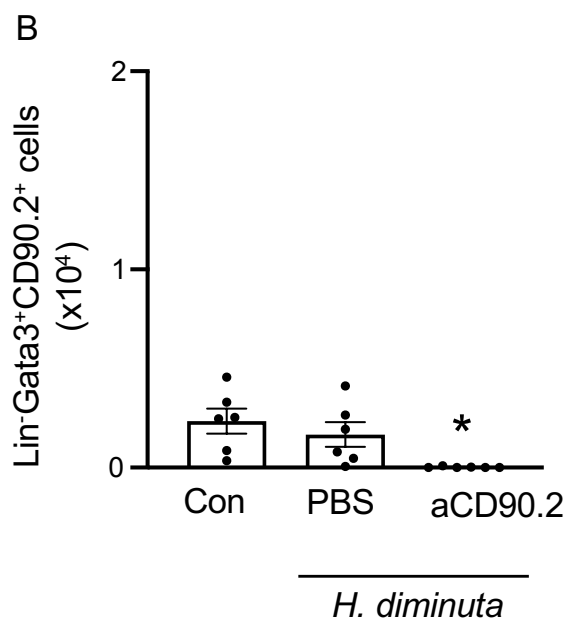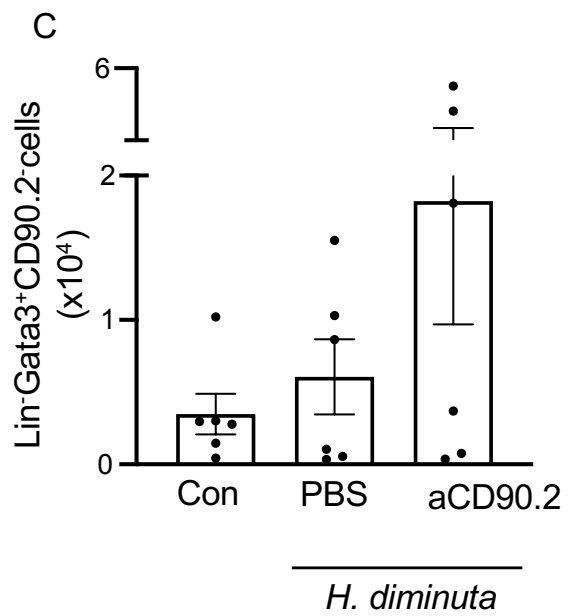

Supplement: S3 Fig — (A) Upon necropsy, cell populations from the jejunal lamina propria were inspected for Gata-3 and CD90.2 expression by flow cytometry, pre-gated as lineage- live lymphocytes. (B) Anti-CD90.2 treatment significantly depleted the number of CD90.2+ Gata-3+cells compared to isotype/PBS controls, but did not deplete (C) a population of CD90.2- Gata-3+ cells. Data are mean ± SEM, n = 6/group, pooled from 2 experiments, * p<0.05 analysed by Brown’s Forsyth ANOVA with Dunnett’s test compared to control (Con). (PDF) [file ppat.1012381.s003.pdf]

A

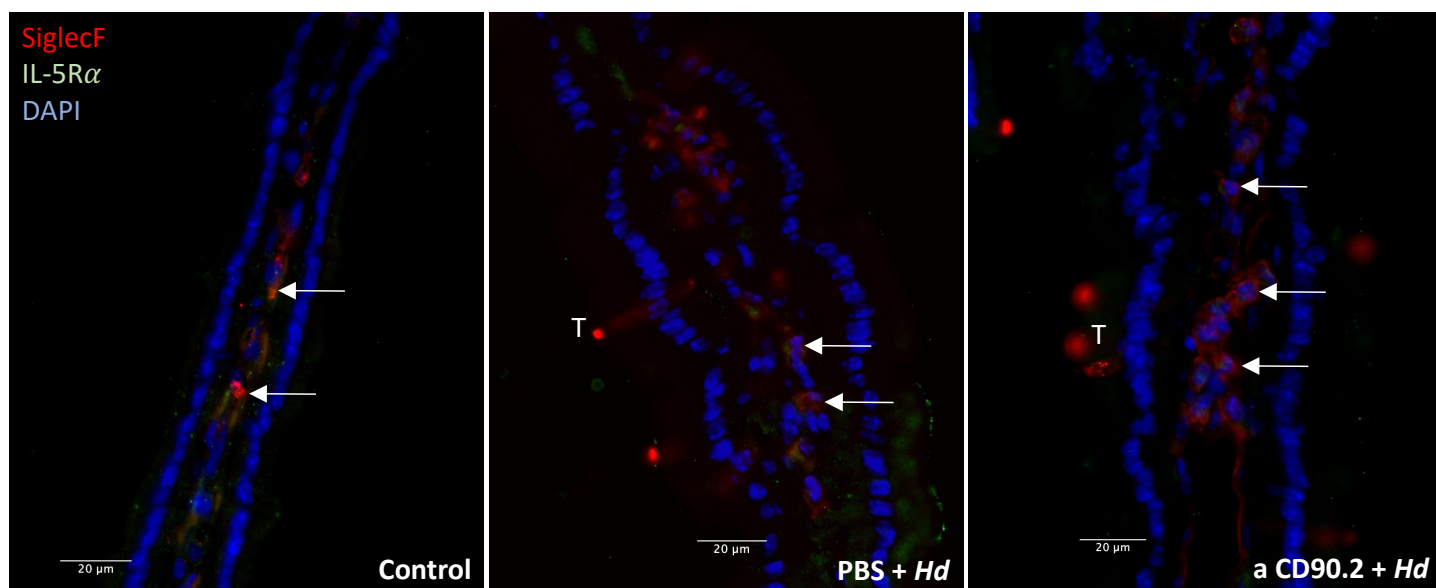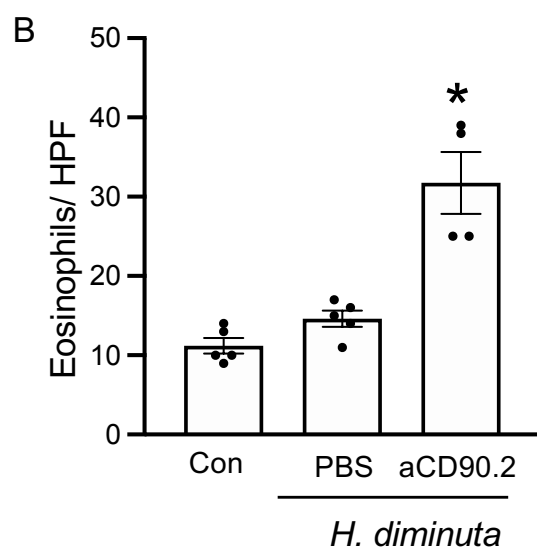

Supplement: S4 Fig — Male Rag-1-/- mice (with and without anti-CD90.2 treatment), were infected with 5 cysticercoids of H. diminuta and assessed at 11 days post infection (dpi). (A) Representative images of mid-jejunal cryosections stained with anti-SiglecF antibody, anti-IL-5Rα antibody and DAPI. “T” stands for tuft cells; white arrows point towards eosinophils (B) Eosinophils enumerated using QuPath for SiglecF+ cell detection over 2–3 high power fields of view (HPF) photographs taken at 40x objective. Data are mean ± SEM, n = 4-5/group, pooled from 3 experiments, * p<0.05 compared to control mice analysed by Brown-Forsythe ANOVA with Dunnett’s post-test for multiple comparison. (PDF) [file ppat.1012381.s004.pdf]

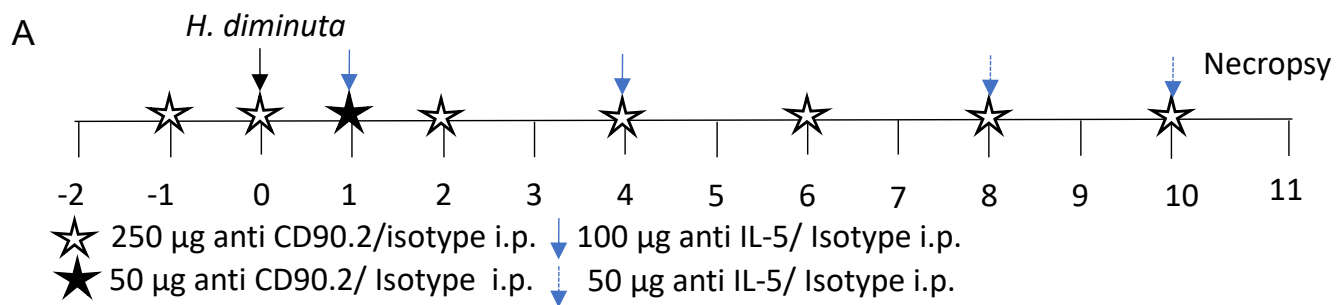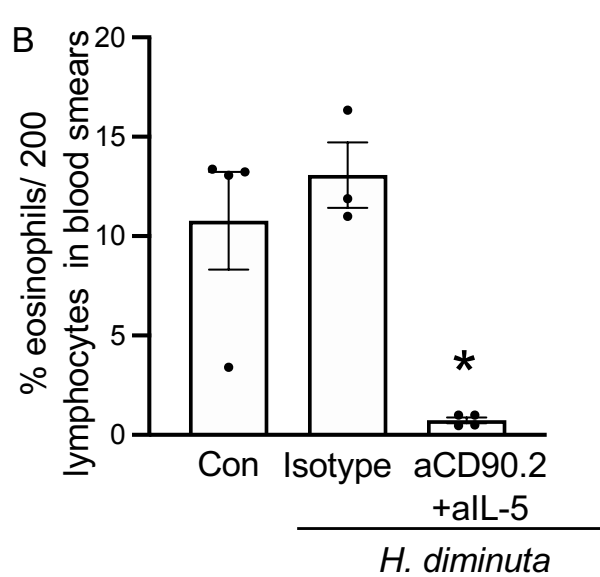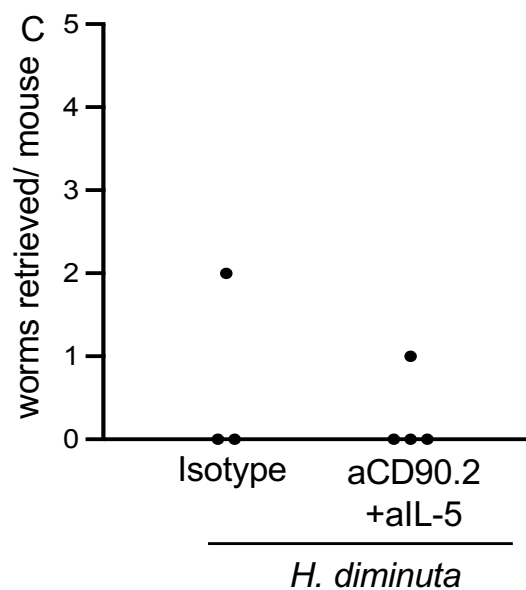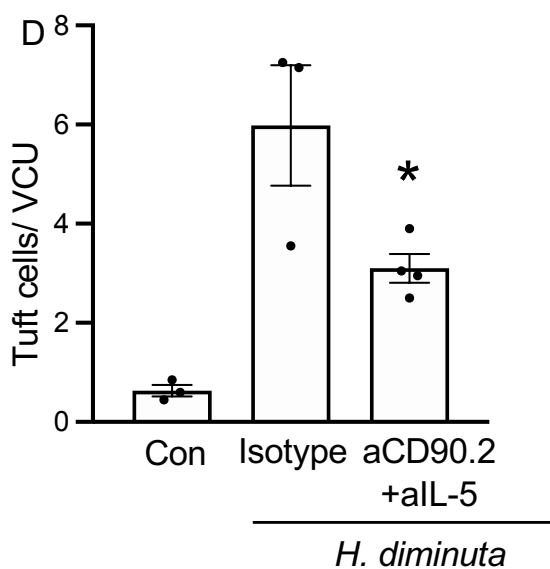

Supplement: S5 Fig — (A) Male Rag-1-/- mice (± anti-CD90.2 and anti-IL-5 antibody treatment) were infected with 5 cysticercoids of H. diminuta and assessed at 11 days post infection (dpi). (B) Blood eosinophil percentage analysed on Giemsa-stained peripheral tail vein blood smears show marked reduction in the anti-IL-5 treated group. (C) Small intestines were flushed with ice-cold PBS and contents observed under a dissection microscope for worms. (D) Tuft cells enumerated per VCU (Villus Crypt Unit). Data are mean ± SEM, n = 3-4/group, * p<0.05 compared to controls (Con), analysed by (B) Kruskal Wallis test with Dunn’s post-test or (D) Brown-Forsythe and Welch’s ANOVA with Dunnett’s post-test for multiple comparisons. (PDF) [file ppat.1012381.s005.pdf]

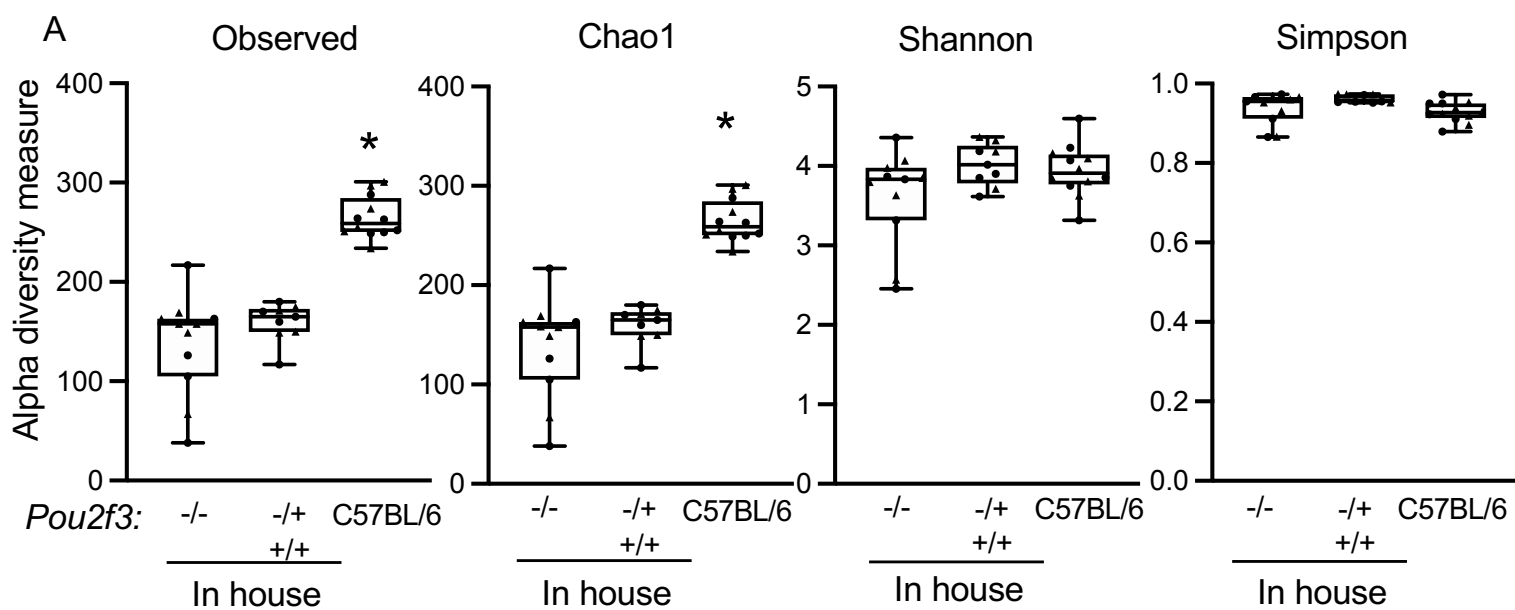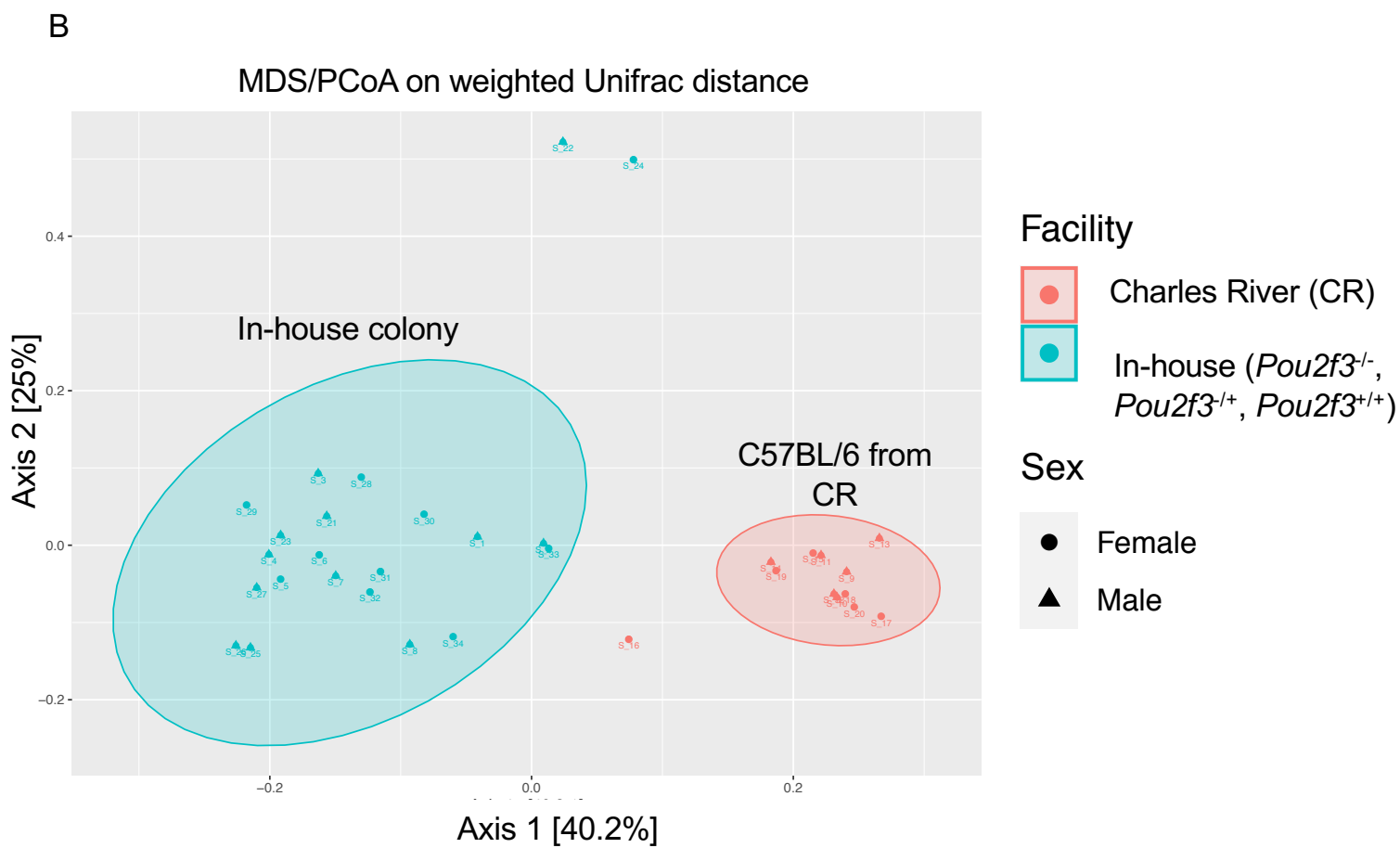

Supplement: S6 Fig — Fecal 16S rRNA sequencing was conducted on samples from female (●) and male (▲) C57BL/6 mice commercially purchased from Charles River (CR), homozygous Pou2f3-/- mice bred in-house from Pou2f3-/- X Pou2f3-/- parents and littermates (Pou2f3 -/-, -/+ and +/+) bred from Pou2f3-/+/Pou2f3-/- X Pou2f3-/+ parents. (A) α diversity plots reveal significant differences in bacterial compositions of mice purchased from Charles River in the Observed and Chao1 measures of richness compared to in-house colonies, but a similarity between the in-house breeding colonies in all measures. Data are box and whisker plots: horizontal line at median, box plots show 25–75% quartiles and vertical line,—minimum and maximum value, n = 9–11, * p<0.05 analysed by Kruskal Wallis test with Dunn’s multiple comparison test compared to Pou2f3-/- mice. (B) β diversity (PCoA, Weighted unifrac distance) shows separated clustering of colonies of commercially purchased mice (grouped to the right, in red) and colonies bred in-house (grouped to the left, in blue). (PDF) [file ppat.1012381.s006.pdf]

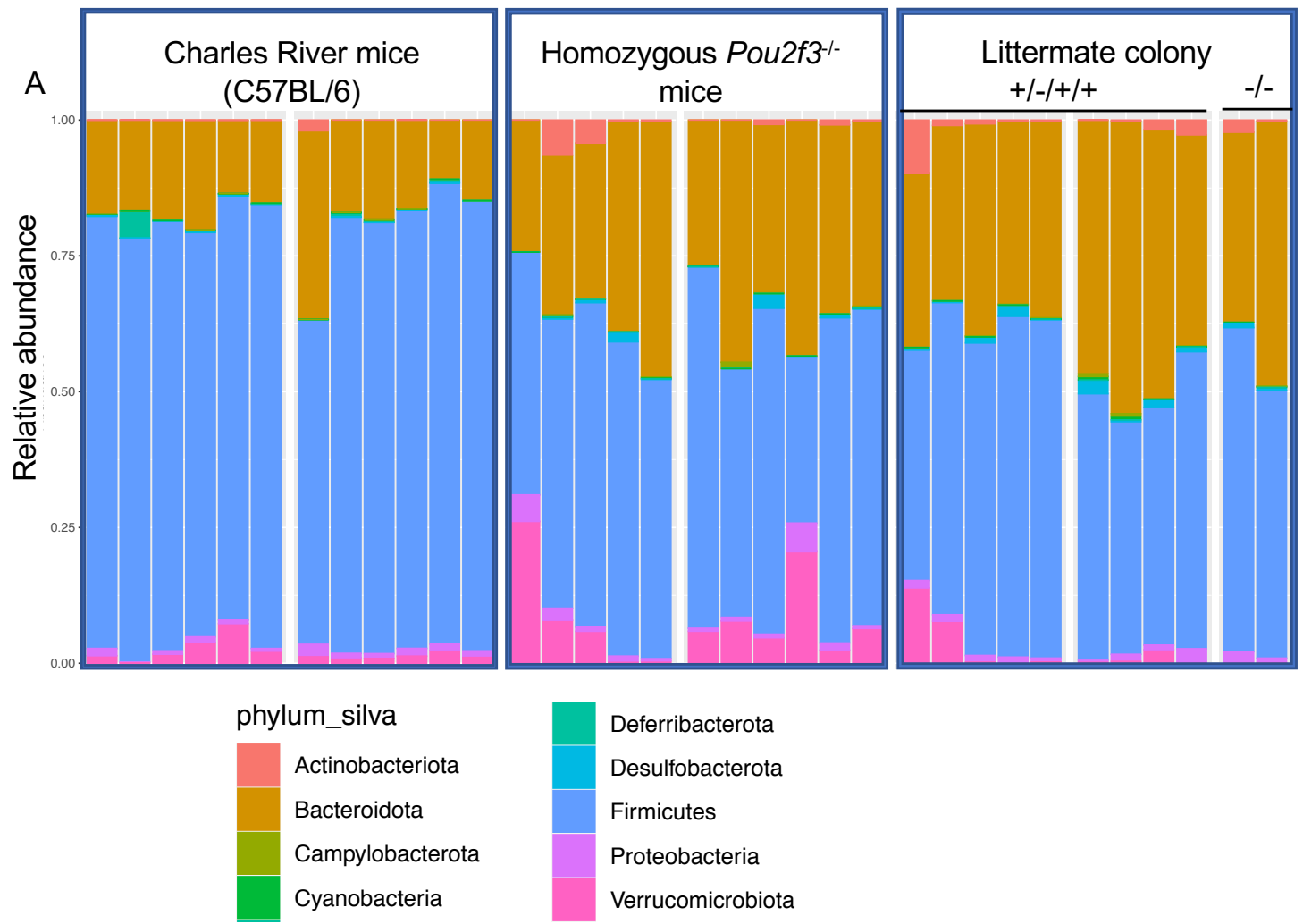

Supplement: S7 Fig — Fecal 16S rRNA sequencing was conducted on samples from female and male C57BL/6 mice commercially purchased from Charles River (CR), homozygous Pou2f3-/- mice from Pou2f3-/- X Pou2f3-/- parents and littermates (Pou2f3 -/- or -/+ and +/+) bred from Pou2f3-/+/-/- X Pou2f3-/+ parents in-house. (PDF) [file ppat.1012381.s007.pdf]

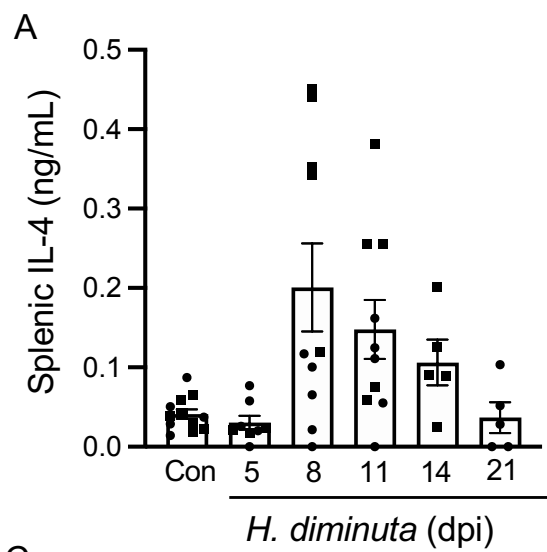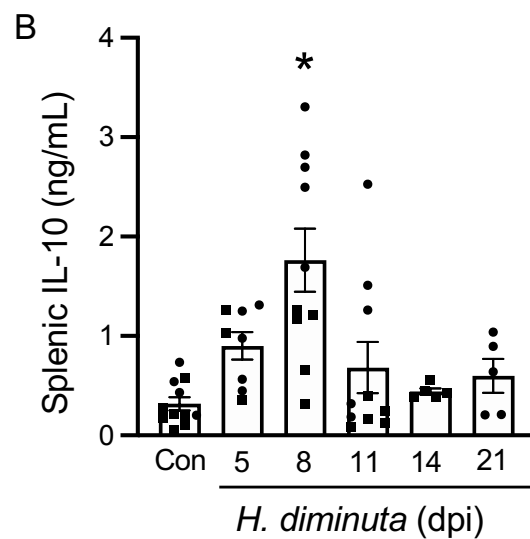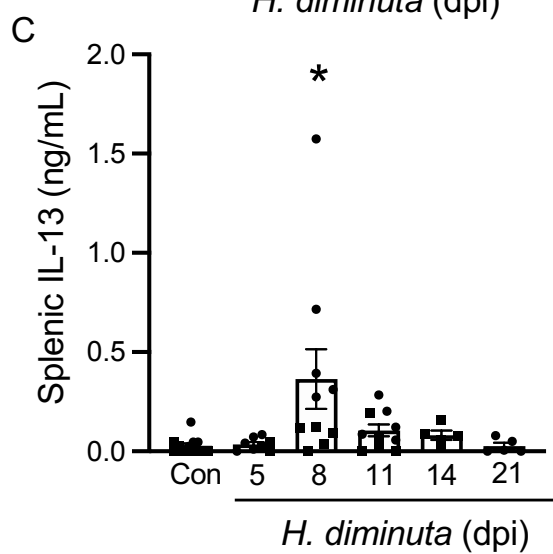

Supplement: S8 Fig — Male (●) and female (■) Pou2f3-/- mice were infected with 5 cysticercoids of H. diminuta and assessed at days post-infection (dpi). Cytokine ELISAs for IL-4, -10, and -13 were performed on supernatants from splenic cells (5x10^6^) stimulated with concanavalin A (2 μg/mL) for 48h. Data are mean ± SEM, n = 5-10/group, pooled from 2–3 experiments, * p<0.05 compared to uninfected mice (Con) analysed by Kruskal Wallis Test and Dunn’s post-test. (PDF) [file ppat.1012381.s008.pdf]

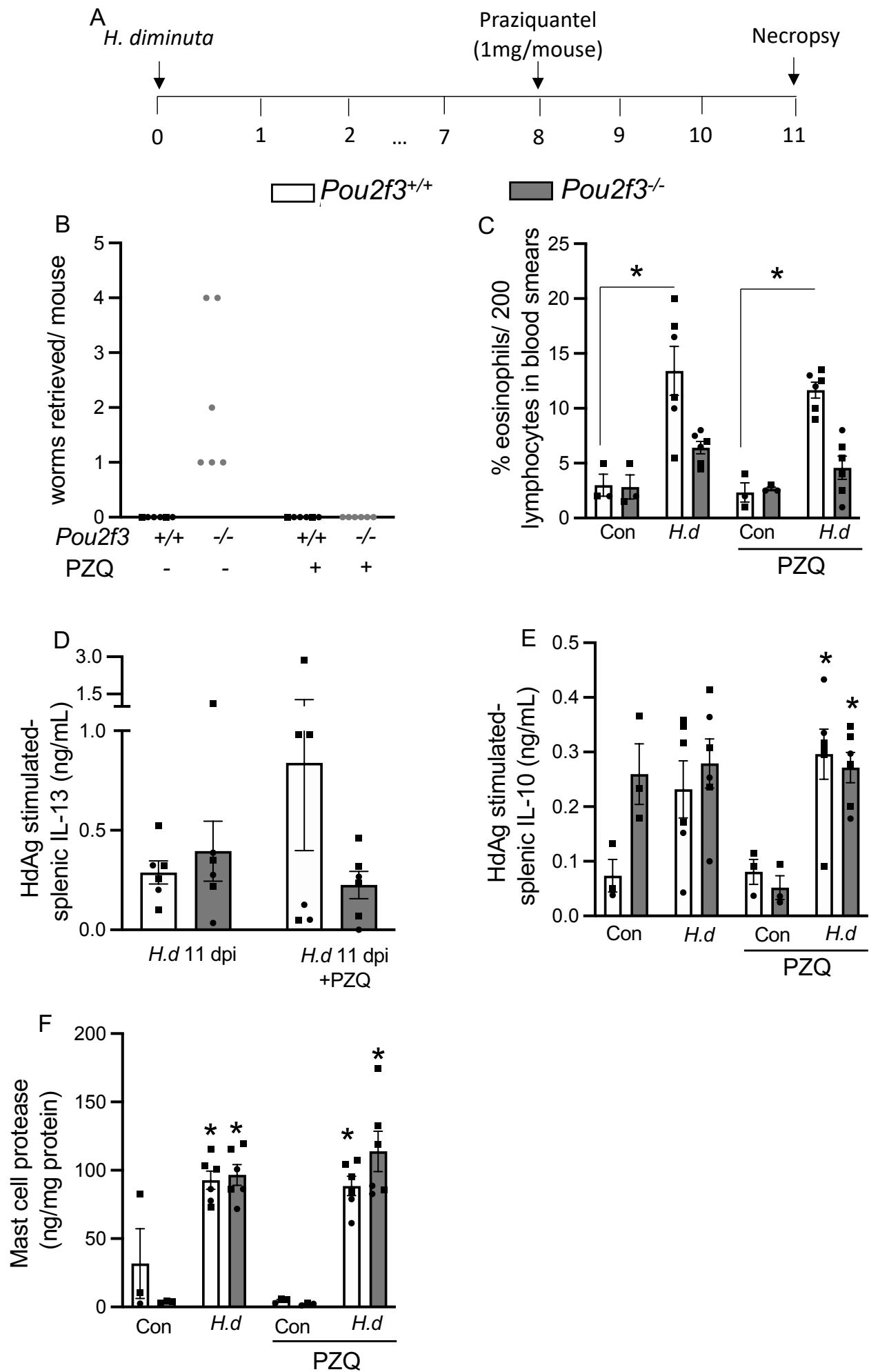

Supplement: S9 Fig — (A) Male (●) and female (■) Pou2f3-/- and C57BL/6 mice were infected with 5 cysticercoids of H. diminuta, treated with PZQ (1 mg/mouse by oral gavage) at 8 days post-infection (dpi) and assessed at 11 dpi. (B) Small intestines were flushed with ice-cold PBS and contents observed under a dissection microscope for worms. (C) Blood eosinophil percentage was analysed on Giemsa-stained peripheral tail vein blood smears. (D, E) Cytokine ELISAs for IL-13 and IL-10 were performed on supernatants from splenic cells (5x10^6^) stimulated with a PBS-soluble crude extract of adult H. diminuta (HdAg, 200 μg/mL, 96h). (F) Mast cell protease-1 concentrations were measured by ELISA in mid-jejunal homogenates. (PDF) [file ppat.1012381.s009.pdf]

A

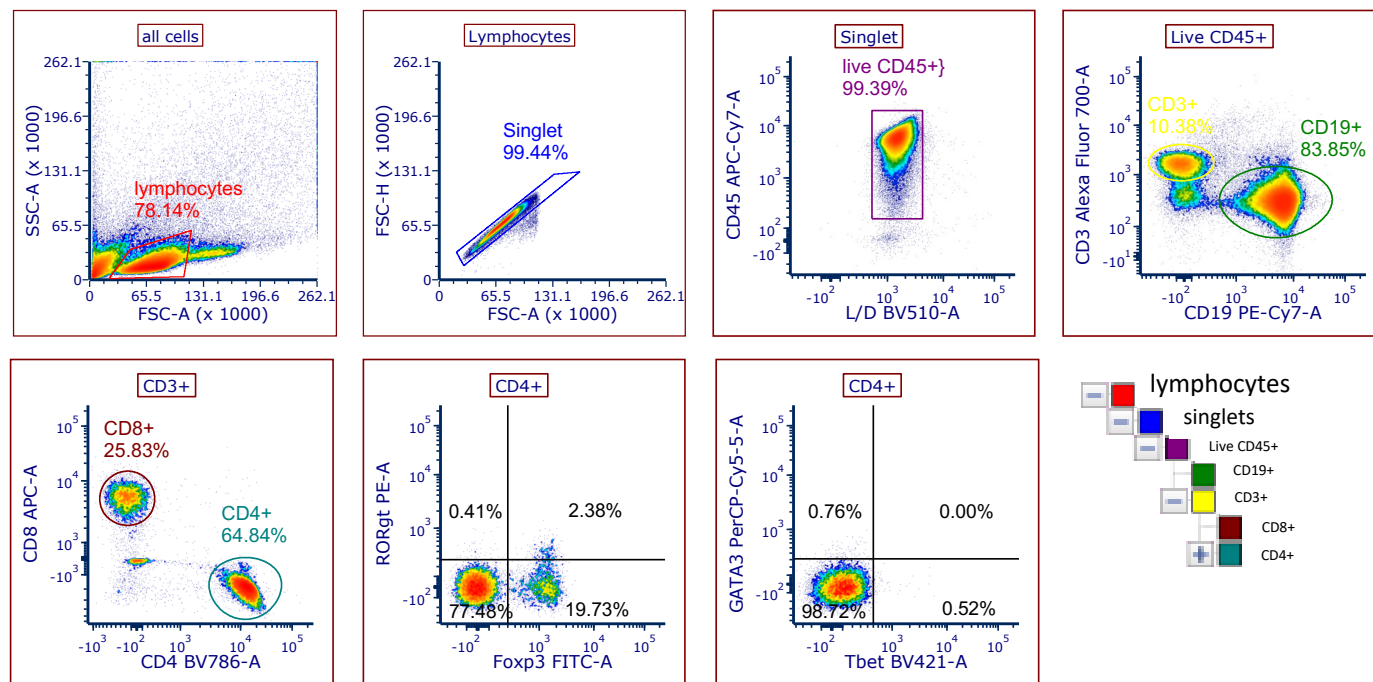

□ *Pou2f3*<sup>+/+</sup>

■ *Pou2f3*<sup>-/-</sup>

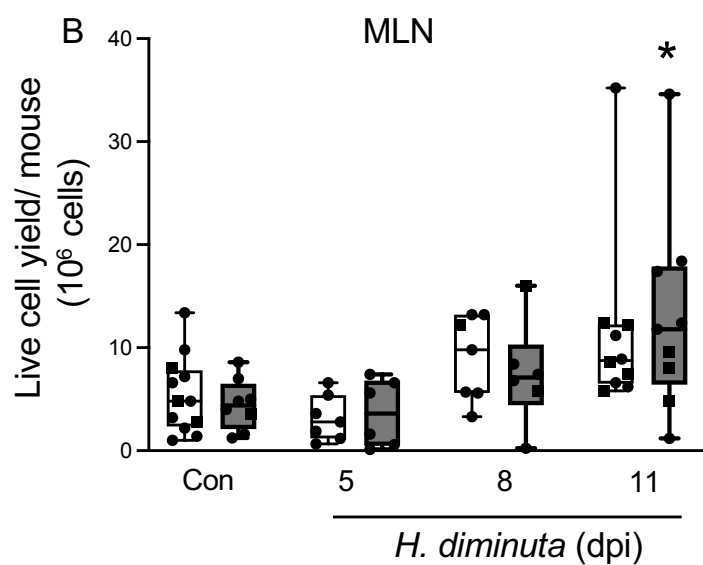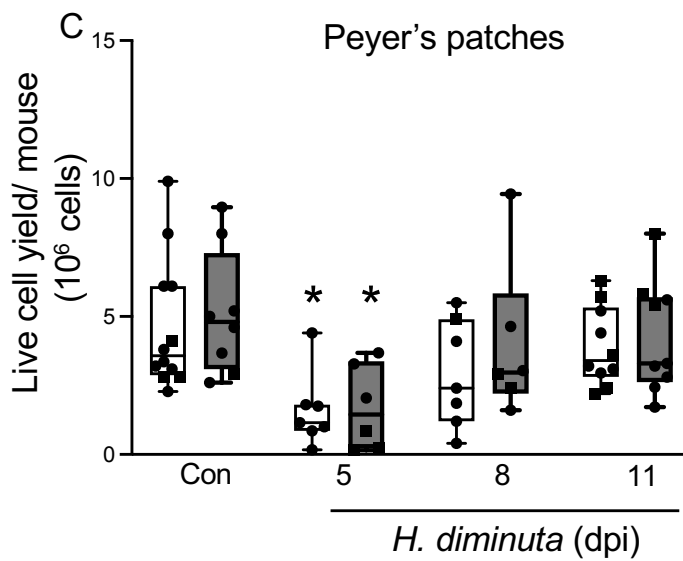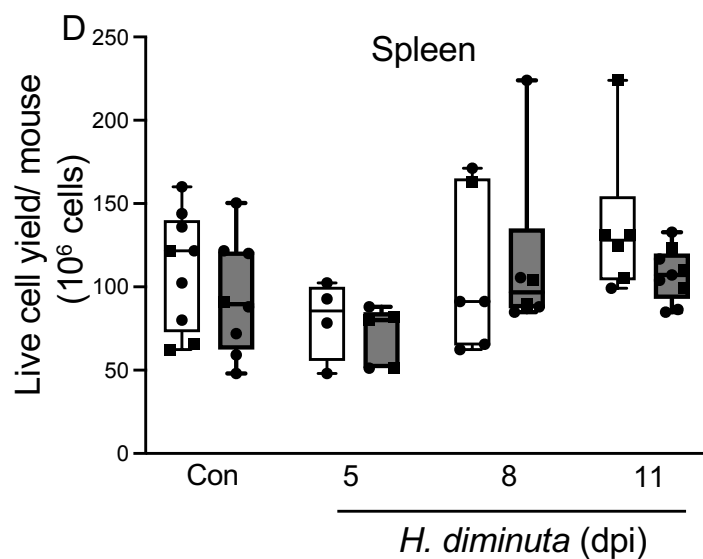

Supplement: S10 Fig — Male (●) and female (■) littermate Pou2f3+/+, +/-, -/- mice were infected with 5 cysticercoids of H. diminuta and assessed at days post-infection (dpi). Live lymphocyte populations were analysed by flow cytometry using single cell suspensions isolated from the (B) mesenteric lymph nodes (MLN), (C) Peyer’s patches (without ConA stimulation) and (D) spleen. Data are mean ± SEM, n = 5-12/group, pooled from 2–3 experiments, * p<0.05 compared to uninfected mice (Con) of each genotype, analysed by Kruskal Wallis test and Dunn’s post-test. (PDF) [file ppat.1012381.s010.pdf]

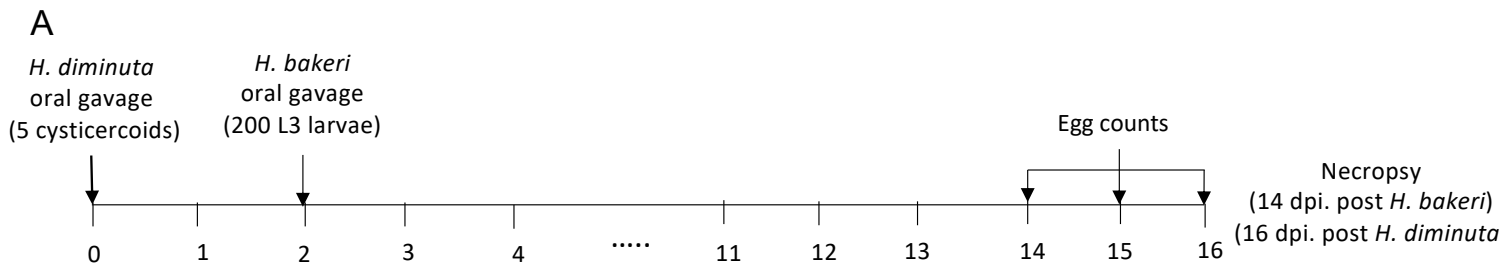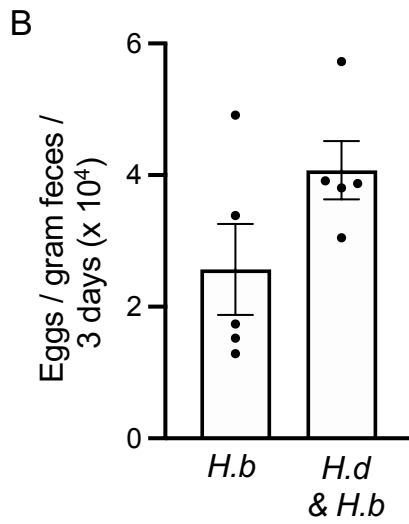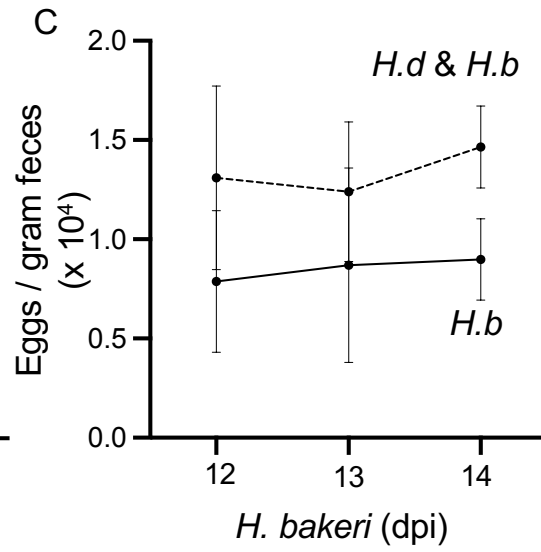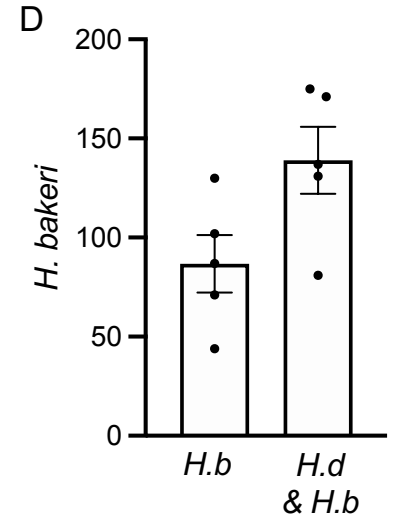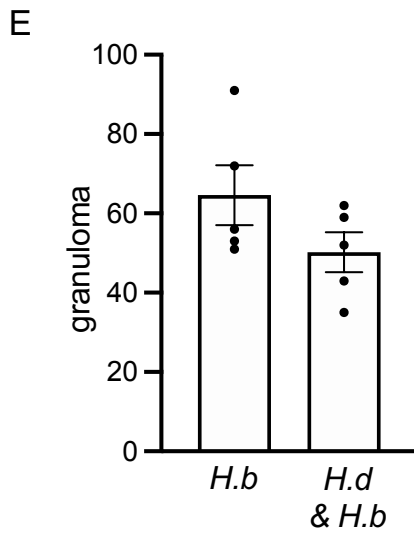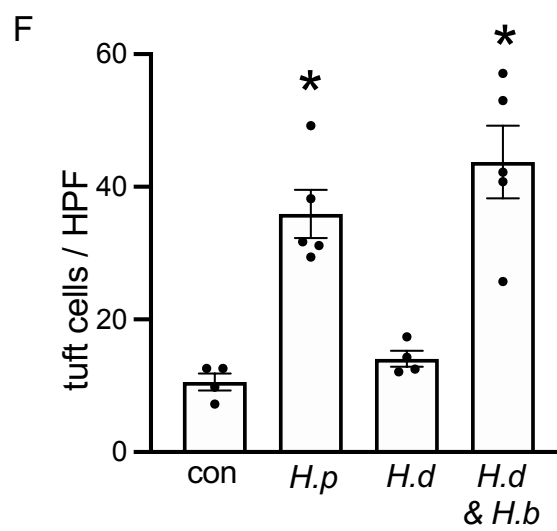

Supplement: S11 Fig — (A) Male C57BL/6 mice were infected with 5 cysticercoids of H. diminuta (H. d.), then 200 L3 of H. bakeri (H.b.) and necropsied at 16 days post-infection with H. diminuta. Single parasite-infected and naïve mice served as controls. (B-E) Co-infected mice showed no significant differences in H. bakeri egg output, luminal worms, or granulomas. (F) DCLK1+ tuft cells were enumerated per high power field (HPF) of view (40x objective) in swiss rolls made from the first 10 cm of small intestine. Data are as mean ± SEM, n = 5/group, * p <0.05 compared to H. bakeri only mice or control (con) uninfected mice by (A-E) Welch’s unpaired t test or (F) Browns Forsythe and Welch’s ANOVA test and Dunnet’s post-test for multiple comparisons. (PDF) [file ppat.1012381.s011.pdf]
