# Supplementary material for: Enteric tuft cells coordinate timely expulsion of the tapeworm Hymenolepis diminuta from the murine host by coordinating local but not systemic immunity
Source: PLoS Pathog. 2024 Jul 31;20(7):e1012381. doi: 10.1371/journal.ppat.1012381 (PMC11290655; doi:10.1371/journal.ppat.1012381)
Supplement: S1 Table — (PDF) [file ppat.1012381.s012.pdf]

S1 Table. Comparison of uninfected control and *H. diminuta*-infected C57BL/6 wild-type and *Pou2f3*$^{-/-}$ littermates. Data are mean ± SEM; dpi, days post-infection; *H. diminuta* at 5 cysticercoids by oral gavage; multiple unpaired T tests with Welch's correction and Hom Sidak test.

	WT Pou2f3$^{+/-}$ and $^{+/+}$	*Pou2f3*$^{-/-}$
Serum IgG1 (µg/mL)		
Control (n=4-5)	2.6 ± 0.4	1.4 ± 0.3
H. diminuta 8 dpi (n=7)	2.5 ± 0.8	3.0 ± 0.5
Serum IgG2α (µg/mL)		
Control (n=4-5)	3.1 ± 0.9	4.6 ± 3.1
H. diminuta 8 dpi (n=9)	1.1 ± 0.3	2.7 ± 1.4
Colonic motility (time to expel 3 mm bead (seconds))		
Control (n=3-6)	86 ± 16	64 ± 6
H. diminuta 8 dpi (n=3)	105 ± 8	116 ± 19
Ussing chamber analysis		
Baseline short circuit current (Isc, µA/cm^2)		
Control (n=3-5)	66 ± 30	74 ± 18
H. diminuta 8 dpi (n=3-4)	68 ± 12	54 ± 10
Carbachol (100 µM) (ΔIsc µA/cm^2)		
Control (n=3-5)	68 ± 6.5	65 ± 14
H. diminuta 8 dpi (n=3-4)	37 ± 4	103 ± 23
Forskolin (10 µM) (ΔIsc µA/cm^2)		
Control (n=3-5)	84 ± 10	93 ± 5
H. diminuta 8 dpi (n=3-4)	70 ± 9	88 ± 25
