# Supplementary material for: Enteric tuft cells coordinate timely expulsion of the tapeworm Hymenolepis diminuta from the murine host by coordinating local but not systemic immunity
Source: PLoS Pathog. 2024 Jul 31;20(7):e1012381. doi: 10.1371/journal.ppat.1012381 (PMC11290655; doi:10.1371/journal.ppat.1012381)
Supplement: S2 Table — (PDF) [file ppat.1012381.s013.pdf]

S2 Table. Immunophenotyping of uninfected *and H. diminuta*-infected wild-type (WT; *Pou2f3*$^{+/+}$ and *Pou2f3*$^{+/-}$) and tuft cell-deficient *Pou2f3*$^{-/-}$ C57BL/6 mice. Data are Mean ± SEM, * $p < 0.05$, analysed by Two Way ANOVA and Dunnett's post test compared to uninfected controls.

Region	Cell type (number of cells)	Genotype	Control (n=7-10)	5 dpi. (n=5)	8 dpi. (n=6)	11 dpi. (n=9-10)
MLN	B cells (CD19$^+$) (x 10^6)	WT	1.7±0.5	0.9±0.4	2.5±0.6	3.5±1
		Pou2f3$^{-/-}$	0.9±0.2	0.8±0.3	2.5±0.8	4.9±1.5*
	Cytotoxic T cells (CD8$^+$) (x 10^5)	WT	8.9±1.9	5.9±1.9	15.8±3.6	22.1±5.9*
		Pou2f3$^{-/-}$	7±1.6	9.3±3.4	13.2±4	24.9±6.1*
	T helper cells (CD4$^+$) (x 10^6)	WT	1.4±0.3	0.7±0.3	2.3±0.5	3.3±0.8*
		Pou2f3$^{-/-}$	0.9±0.2	1.2±0.5	1.6±0.5	3±0.7*
	Tbet$^+$ CD4$^+$ T cells (x 10^4)	WT	1.5±0.4	1.6±0.9	1.9±0.6	5±0.8*
		Pou2f3$^{-/-}$	1±0.2	1.1±0.4	1.7±0.6	5.4±2*
	RORɣT$^+$ CD4$^+$ T cells (x 10^3)	WT	5.9±1.7	6.1±2	8.8±3.9	12.7±2.9
		Pou2f3$^{-/-}$	3.7±1.2	11.2±4.4	7.7±2.1	9.3±2.7
Peyer's patches	B cells (CD19$^+$) (x 10^6)	WT	2.8±0.5	0.9±0.3*	1.8±0.4	2.3±0.3
		Pou2f3$^{-/-}$	2.9±0.5	1.1±0.5*	2.4±0.8	2.3±0.4
	Cytotoxic T cells (CD8$^+$) (x 10^5)	WT	1.3±0.3	0.5±0.2	0.9±0.3	1.1±0.2
		Pou2f3$^{-/-}$	1.2±0.2	0.5±0.2	1.2±0.4	1.5±0.4
	T helper cells (CD4$^+$) (x 10^6)	WT	0.4±0.1	0.2±0.1*	0.3±0.1	0.4±0.1
		Pou2f3$^{-/-}$	0.4±0.1	0.2±0.1	0.4±0.1	0.4±0.1
	Tbet$^+$ CD4$^+$ T cells (x 10^4)	WT	0.5±0.2	0.2±0.2	0.3±0.2	0.7±0.2
		Pou2f3$^{-/-}$	0.6±0.2	0.8±0.4	0.9±0.4	0.7±0.3
	RORɣT$^+$ CD4$^+$ T cells (x 10^3)	WT	5.2±1.5	1.4±0.5	2.9±1.3	4.5±0.8*
		Pou2f3$^{-/-}$	4.3±0.7	1.9±0.8	3.9±0.8	1.7±0.5
Spleen	B cells (CD19$^+$) (x 10^6)	WT	44.6±5.7	30.4±6.1	38.3±6.8	50.2±8.7
		Pou2f3$^{-/-}$	40.5±5.8	26±5.2	26±5.2	37.8±2.5
	Cytotoxic T cells (CD8$^+$) (x 10^5)	WT	76.5±7.5	65.9±14.1	89.8±16.7	112.9±19.2
		Pou2f3$^{-/-}$	114.4±27.8	52±9.9	52±9.9	85.9±7.2
	T helper cells (CD4$^+$) (x 10^6)	WT	12.7±1.3	10±2.2	14.3±3.3	19.4±3.6
		Pou2f3$^{-/-}$	13.4±2.2	7±1.6	7±1.6	12.4±2
	Gata3$^+$ CD4$^+$ T cells (x 10^4)	WT	1.6±0.4	0.9±0.4	5.4±1.6	2.8±0.5
		Pou2f3$^{-/-}$	2.1±0.6	0.8±0.3	0.8±0.3	0.9±0.4
	Foxp3$^+$ CD4$^+$ T cells (x $10^{5)}$	WT	17.4±2.1	12.9±3	17.2±3.8	23.5±3.9
		Pou2f3$^{-/-}$	18.5±4.8	8.8±2	8.8±2	13.6±2.3
	Tbet$^+$ CD4$^+$ T cells (x 10^4)	WT	18.5±8.6	17.6±4.6	21.6±2.8	16.4±4.4
		Pou2f3$^{-/-}$	22.3±5.2	6.3±1.3	6.3±1.3	10±1.7
	RORɣT$^+$ CD4$^+$ T cells (x 10^3)	WT	10.2±3.7	18.2±8.6	13.1±4.7	10.7±3.9
		Pou2f3$^{-/-}$	17.7±5.3	12.8±6	12.8±6	5.7±1.8
