# Supplementary material for: Enteric tuft cells coordinate timely expulsion of the tapeworm Hymenolepis diminuta from the murine host by coordinating local but not systemic immunity
Source: PLoS Pathog. 2024 Jul 31;20(7):e1012381. doi: 10.1371/journal.ppat.1012381 (PMC11290655; doi:10.1371/journal.ppat.1012381)
Supplement: S3 Table — (PDF) [file ppat.1012381.s014.pdf]

S3 Table. Primers used for genotyping or RT-PCR on murine cells

Gene target	Forward primer	Reverse primer
WT_*Pou2f3*/KO_*Pou2f3*_LacZ genotyping	ggagaagtcggctcctctcccattatc/ gcattctagttgtggtttgtcc	cagtcaatagctggggagctgaagctc
18S	atggccgttcttagttggtg	cgctgagccagtcagtgtag
Dclk1	actaaggacagggcacagac	tgaccggggaaagcagtaat
Il-25	tggcttagtcatgctcacca	gtttctccccaagtcctcca
Alox5	ggcccgagatgaccaaattc	aactggatatctcggggcag
Trpm5	gacatggccgagttcttgac	aagtctttgagtacgcggga
