# Supplementary material for: Enteric tuft cells coordinate timely expulsion of the tapeworm Hymenolepis diminuta from the murine host by coordinating local but not systemic immunity
Source: PLoS Pathog. 2024 Jul 31;20(7):e1012381. doi: 10.1371/journal.ppat.1012381 (PMC11290655; doi:10.1371/journal.ppat.1012381)
Supplement: S4 Table — (PDF) [file ppat.1012381.s015.pdf]

S4 Table. List of antibodies used for flow cytometry and immunohistochemistry

| Flow cytometry | | | |
|---|---|---|---|
| Antibody target- Fluorescent marker | Clone | Company | Working concentration |
| CD16/32 | 93 | Biolegend | 1 µg/$10^6$ cells |
| CD4-BV786 | RM4-5 | BD Biosciences | 1 µg/mL |
| CD19-PE-Cy7 | 6D5 | Biolegend | 1 µg/mL |
| CD45-APC-Cy7 | 30-F11 | Biolegend | 1 µg/mL |
| CD3-AF700 | 145-2C11 | Biolegend | 2.5 µg/mL |
| CD8α-APC | 53-6.7 | Biolegend | 1 µg/mL |
| T-bet-BV421 | O4-46 | BD Biosciences | 2 µg/mL |
| FoxP3-FITC | FJK-16s | eBioscience | 2.5 µg/mL |
| RORɣt-PE | B2D | eBioscience | 2 µg/mL |
| RORgt-APC | B2D | eBioscience | 2 µg/mL |
| GATA3-PE | TWAJ | eBioscience | 0.1 µg/mL |
| GATA3-PerCP-Cy5.5 | 16E10A23 | Biolegend | 0.1 µg/mL |
| CD90.2-BV421 | 53-2.1 | eBioscience | 0.25 µg/mL |
| **Lineage markers (ILC gating)** | | | |
| Ly6G-FITC | 1A8 | eBioscience | 5 µg/mL |
| B220-FITC | RA3-6B2 | eBioscience | 5 µg/mL |
| NK1.1-FITC | PK136 | eBioscience | 5 µg/mL |
| CD3-FITC | 17A2 | eBioscience | 5 µg/mL |
| CD8-FITC | 53-6.7 | eBioscience | 5 µg/mL |
| **Immunohistochemistry** | | | |
| Primary antibody target, host species it was raised in | Clone/ (#) catalogue number | Company | Concentration |
| DCLK1, rabbit | # ab31704 | Abcam | 1 µg/mL |
| SiglecF, rat | S17007L | Biolegend | 2.5 µg/mL |
| IL-5R$\alpha$, rabbit | #BS-2601R | ThermoFisher Scientific | 10 µg/mL |
| Secondary antibody - fluorescent marker | Clone/ (#) catalogue number | Company | Concentration |
| Donkey anti-rabbit IgG-Cy3 | #711-165-152 | Jackson ImmunoResearch | 3 µg/mL |
| Donkey anti-rat IgG-Cy5 | #712-175-153 | Jackson ImmunoResearch | 3 µg/mL |
